# Is Targeting Nerve Growth Factor Antagonist a New Option for Pharmacologic Treatment of Low Back Pain? A Supplemental Network Meta-Analysis of the American College of Physicians Guidelines

**DOI:** 10.3389/fphar.2021.727771

**Published:** 2021-08-31

**Authors:** Ziqin Cao, Qiangxiang Li, Jia Guo, Yajia Li, Jianhuang Wu

**Affiliations:** ^1^ Department of Spine Surgery and Orthopaedics, Xiangya Hospital, Central South University, Changsha, China; ^2^ Ningxia Geriatric Disease Clinical Research Center, People’s Hospital of Ningxia Hui Autonomous Region, Yinchuan, China; ^3^ National Clinical Research Center for Geriatric Disorders of Xiangya Hospital, Central South University, Yinchuan, China; ^4^ Department of Hunan Institute of Geriatrics, Hunan People’s Hospital, Changsha, China; ^5^ Department of Dermatology, Xiangya Hospital, Central South University, Changsha, China; ^6^ National Clinical Research Center for Geriatric Disorders, Xiangya Hospital, Central South University, Changsha, China

**Keywords:** targeting nerve growth factor antagonist, low back pain, pharmacological treatment, network meta-analysis, comparative efficacy and safety 3

## Abstract

**Objective:** It has been found that targeting nerve growth factor antagonists (ANGF) have excellent effects in the treatment of chronic pain, and the current pharmacologic treatments have very limited effects on low back pain (LBP). Thus we conducted this network meta-analysis (NMA) to study the efficacy and safety of ANGF for the treatment of LBP, and to guide for clinical practice and further research.

**Method:** PubMed, Scopus, Embase, CNKI, and the Cochrane Library were searched from January 1980 to March 2021. A frequentist framework network meta-analysis with a random-effect model was performed. Ranking effects were calculated by surface under the cumulative ranking analysis (SUCRA) and clusterank analysis.

**Results:** This NMA identified 30 studies, involving 9,508 patients with LBP. ANGF reported both superior effect on pain relief {SUCRA 82.1%, SMD 0.89, 95% CI [(0.26,1.51)]} and function improvement {SUCRA 77.3%, SMD 0.93, 95% CI [(0.27,1.58)]} than placebo, and did not showed any higher risk of treatment-emergent adverse effects {RR 1.11, 95% CI [(0.97,1.27)]} or serious adverse effects {RR 1.03, 95% CI [(0.54,1.97)]}, but it was associate with a special risk of rapidly progressive osteoarthritis. ANGF displayed the greatest potential to be the most effective and safest treatment (cluster-rank value for function improvement and safety: 4266.96, for pain relief and safety: 4531.92).

**Conclusion:** ANGF could relieve pain and improve function effectively and are superior to other traditional drugs recommended by guidelines. Although no significant difference in tolerability and safety between ANGFs and placebo was found, the rapid progression of original osteoarthritis which may be related to the use of ANGFs still needs special attention and furtherly verification by clinical trials.

**Systematic Review Registration**: PROSPERO, identifier [CRD42021258033].

## Introduction

Low back pain (LBP), defined as the pain occurring between the lower edge of the rib cage and the hip, is a common orthopedic symptom in all age groups. LBP may be of nociceptive, neuropathic, or mixed origin. It is associated with complex pathological causes, including vertebral fractures, malignant tumors, infections, disc herniation, facet pathology, and others. LBP is a leading cause of disability worldwide and results in a considerable health burden; disability caused by LBP has increased by 54% globally in the past 20 years (Dionne et al., 2008; GBD, 2016; Buchbinder et al., 2018). Most LBP patients cannot determine the specific source of their pain and are classified as having non-specific LBP (Maher et al., 2017). According to the current guidelines (Foster et al., 2018), non-pharmacological treatments are the recommended first-line treatments, including exercise and rehabilitation training. However, this may often be insufficient for some patients, and additional treatments are needed. The selected prescription drugs that are used for LBP according to the American College of Physicians (ACP)’s Clinical Guidelines (Qaseem et al., 2017) include antidepressants, acetaminophen, nonsteroidal anti-inflammatory drugs (NSAIDs), opioids, skeletal muscle relaxants (SMRs), anticonvulsants (including gabapentin or pregabalin) and others, which were identified to have some effect on LBP. Evidence has revealed that there commonly is an unsatisfactory response to a range of possible drug treatments and carries significantly higher safety risks particularly for long-term treatment (Maher et al., 2017).

Over the past 2 decades, continuing research on nerve growth factor (NGF) and its related molecular targets has provided a new model for treating a range of diseases, especially chronic pain (Chang et al., 2016; Schmelz et al., 2019). Animal studies have demonstrated that NGF inhibitors provide relief for chronic inflammatory pain and cancer pain, and the level of pain relief can be superior to morphine (Koltzenburg et al., 1999; Ro et al., 1999; Sevcik et al., 2005). NGF inhibitors also have been proven to relieve nociceptive pain in many clinical trials (Chang et al., 2016). Markman et al. reported on the efficacy and safety of tanezumab when used to treat chronic non-radicular LBP, and they found that tanezumab was effective against chronic LBP, but the outcome was associated with increased serious adverse events (Markman et al., 2020). However, until recently, studies have not considered treatment responses produced by NGF inhibitors when comparing other drugs. Our previous study that involved 38 randomized controlled trials (RCTs) reported that anti-NGFs (ANGFs) exhibited a significantly better effect on pain relief and functional improvement in osteoarthritis (OA) compared to NSAIDs and opioids (Cao et al., 2020). Considering the current evidence and treatment options, we carried out this network meta-analysis to update and supplement information concerning the assessed efficacy of different drugs, including pain reduction, improvement of physical function, and safety.

## Materials and Methods

### Data Sources and Searches

This research strictly followed the Preferred Reporting Items for Systematic Reviews and Meta-Analyses (PRISMA) guidelines (14). Two authors (QL and YL) systematically searched PubMed, Scopus, embase, CNKI, and the Cochrane Library for articles published between January 1980 and March 2021. The following search strategy was utilized: (“low back pain” OR “back pain” OR “lumbar pain” OR “spine pain” OR “radicular pain”) AND (“drugs” OR “pharmacologic treatment” OR “pharmacologic therapy”) AND (“anti-nerve growth factor monoclonal antibody” OR “targeting nerve growth factor inhibitor” OR “tanezumab” OR “fulranumab” OR “fasinumab”) OR (“nonsteroidal anti-inflammatory drug” OR “NSAIDs” OR “celecoxib” OR “etoricoxib” OR “valdecoxib” OR “nimesulide” OR “meloxicam” OR “lornoxicam” OR “ibuprofen” OR “naproxen” OR “diclofenac” OR “aceclofenac”) OR (“acetanilide antipyretic analgesics” OR “acetaminophen” OR “paracetamol”) OR (“skeletal muscle relaxants” OR “SMR”) OR (“antidepressant” OR “duloxetine” OR “amitriptyline”) OR (“opioid” OR “oxycodone” OR “hydromorphone” OR “oxymorphone” OR “tramadol” OR “tapentadol”). The reference lists of included articles also were searched for potentially eligible studies. No restriction was placed on the publication language.

### Study Selection

A research protocol was drafted previously, based on the PICO (population, intervention, control, and outcomes) principle that included the following criteria.

1) Population: patients with LBP.
2) Intervention: pharmacologic treatments for LBP.
3) Comparison: NGF inhibitors were targeted and other pharmacologic treatments were recommended by ACP guidelines.
4) Outcomes: pain relief, improvement of function, and the incidence of adverse effects (AEs).

Following the PICO format, we included studies with the following characteristics: 1) patients that suffered from LBP; 2) participants were treated only with the target drug during the study; 3) compared two or more different pharmacologic treatments with each other or a placebo; 4) utilized prospective parallel-group RCT designs; and 5) reported at least one of the following primary outcomes: pain relief and incidence of treatment-emergent adverse events (TEAEs).

We excluded studies that exhibited the following characteristics: 1) dose-escalation studies with only one treatment strategy; 2) single-arm studies; 3) animal studies, *in vitro* biomechanical studies, cadaver studies, case-control studies, reviews, systematic reviews, meta-analyses, conference abstracts, letters, and no original study data.

We contacted the corresponding authors of studies that had insufficient data. If no response was received, the study was excluded. We also contacted the corresponding authors of studies that only presented data in figures and not as numeric data in text or tables. If no response was obtained, two authors (JG and JW) independently attempted to ascertain the underlying data from the figures. Studies for which this was not possible were excluded. All disagreements were resolved by discussion.

### Data Extraction and Quality Assessment

The methodological quality and risk of bias for all identified studies were evaluated by two authors (QL and JG) (Higgins et al., 2011). Six risks of bias, including sequence generation, allocation concealment, blinding, incomplete outcome data, selection outcome reporting, and other sources of bias, were evaluated and ranked as low, unclear, or high risk of bias.

After quality assessment, two authors (JW and YL) independently extracted information from the included studies, including first author, publication year, number of participants, mean age, gender, disease type (acute, sub-acute, or chronic), mean follow-up time, and outcomes. If available, data obtained through intention-to-treat analyses were used to avoid the influence of withdrawal bias.

### Outcome Measures

The efficacy endpoints were pain relief and improvement of function. No restriction was placed on the type of questionnaire used to evaluate pain. The Roland-Morris Disability Questionnaire (RMDQ-24) was the preferred questionnaire to evaluate the improvement of function. If that questionnaire was not used, any other functional measurement scale that was used was adopted, including the Oswestry Disability Index (ODI) or Western Ontario and McMaster University arthritis index (WOMAC). To minimize potential bias caused by baseline differences and the impact on the reliability of the results and conclusions, changes from the last follow-up to baseline values (CFB) were used to evaluate the relative efficacy. For studies that did not report a change from the baseline value, the correlation coefficient method recommended by the Cochrane Handbook (Higgins, 2011) was used to calculate the change. Standardized mean differences (SMDs) and 95% CIs were used to eliminate the influence of different measurement units and scales on the results.

The safety endpoints were TEAEs and serious AEs (SAEs). TEAEs were defined as any adverse effects that were considered relevant to target drugs and treatments by researchers. SAEs were defined as any adverse effect that could result in death or life-threatening events, hospitalization, or prolonged period of existing hospitalization, disability or incapacity, and anomaly or congenital disabilities. Risk ratios (RRs) and 95% confidence intervals (CI) were used as measures of relative safety. The numbers needed to treat (NNTs) of safety endpoints were also calculated, and number needed to treat for an additional beneficial outcome (NNTB) and number needed to treat for an additional harmful outcome (NNTH) were used to measure the relative positive and harmful safety outcomes respectively (Veroniki et al., 2019).

### Statistical Analysis

Network-meta-analysis (NMA) is a statistical method that combines direct and indirect evidence to analyze the complex clinical problem. This NMA was conducted using Stata/MP (version 14.0, Stata Corp, College Station, TX, United States). A random-effects, multivariate, meta-regression model was built using a frequentist framework to pool proportional variance-covariance matrix data.

The model fit was assessed using the restricted maximum-likelihood method (White et al., 2012). The Z test for inconsistency and node-split tests were used to check the global and local consistency for each model, respectively. When both tests indicated no significant inconsistency (*p* > 0.05), a consistency model was adopted. If inconsistency was identified, a sensitivity analysis was used to discover the source of the inconsistency. Publication bias within each network was evaluated using a funnel plot and Egger’s test.

Significant publication bias was considered in any network whose funnel plot revealed apparent asymmetry, or Egger’s test resulted in a *p*-value > 0.05. The surface under the cumulative ranking (SUCRA) method was used to rank the relative efficacies and safeties of the included treatments (Rücker and Schwarzer, 2015). Treatment with a higher SUCRA value was considered better (Salanti et al., 2011). Clustered ranking plots were constructed to determine the optimal treatment choice by comparing multiple outcome indicators simultaneously. Because the included studies involved different stages of disease (acute, subacute, or chronic), a subgroup analysis was performed to explore the impact on the results. Similarly, because three different drugs (fasinumab, tanezumab, and fulranumab) were included in the anti-NGF group, a subgroup analysis was conducted to compare the three drugs to determine whether any intra-group differences occurred. Differences between treatments were considered significant when the 95% CI did not contain 1 for RRs or 0 for SMDs. *p* < 0.05 was considered statistically significant. Evidence contribution diagram was used to intuitively reflected the weight of direct and indirect estimates in the entire network.

## Results

### Literature Selection

**FIGURE 1:**
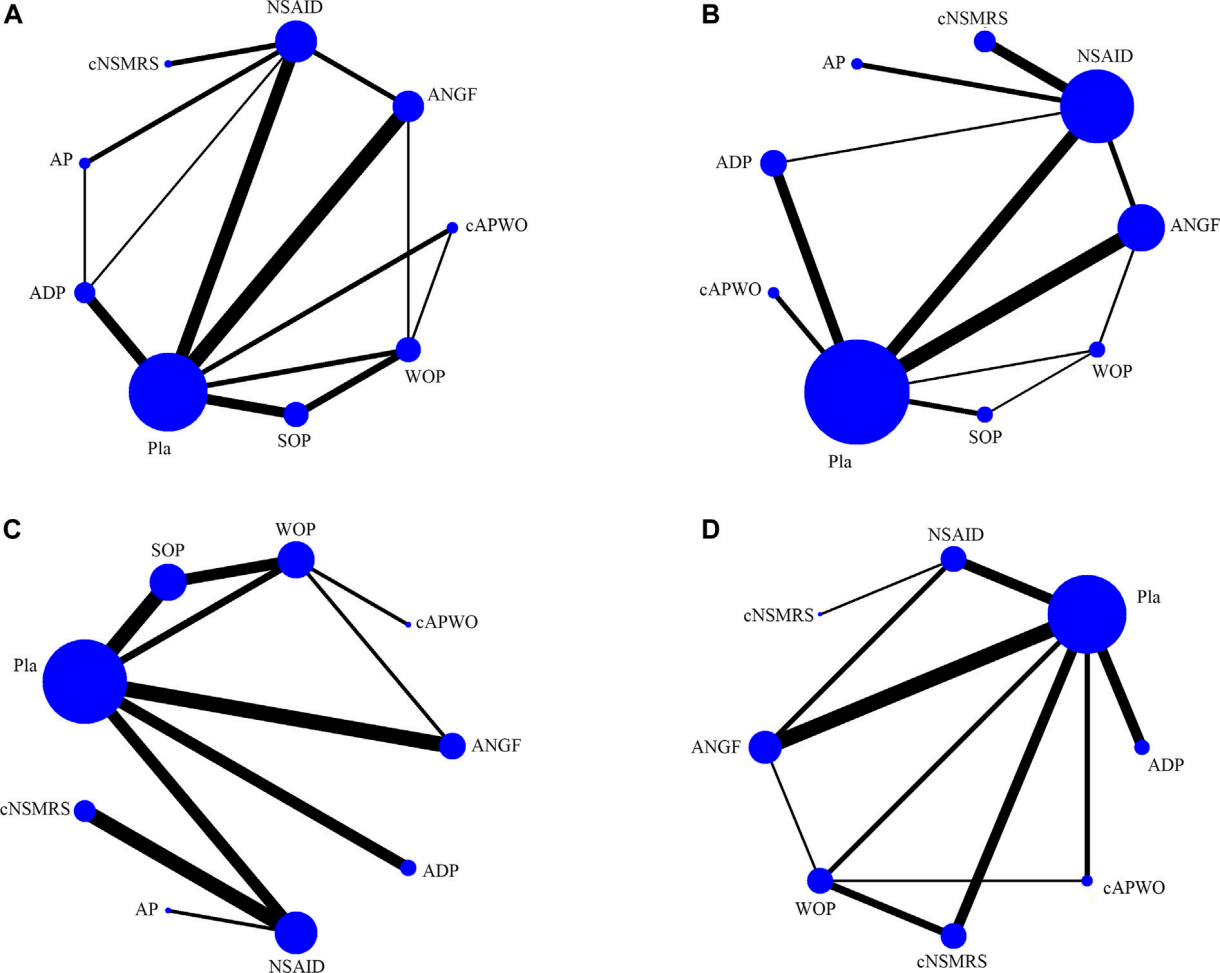
Structure of main network formed by interventions. The lines between treatment nodes indicate the direct comparisons made within randomised controlled trials. The size of the node reflects the number of participants in the intervention, the larger the node, the more participants. **(A)** Pain relief. **(B)** Function improvement. **(C)** TEAEs. **(D)** SAEs.

Thirty studies (Berry and Hutchinson, 1988; Stein et al., 1996; Birbara et al., 2003; Ruoff et al., 2003; Coats et al., 2004; Pallay et al., 2004; Peloso et al., 2004; Kanayama et al., 2005; Perrot et al., 2006; Pareek et al., 2009; Skljarevski et al., 2009; Skljarevski et al., 2010a; Skljarevski et al., 2010b; Buynak et al., 2010; Hale et al., 2010; Katz et al., 2011; Biondi et al., 2013; Kivitz et al., 2013; Rauck et al., 2014; Tiseo et al., 2014; Friedman et al., 2015; Rauck et al., 2015; Baron et al., 2016; Bedaiwi et al., 2016; Gottlieb and Njie, 2016; Konno et al., 2016; Sanga et al., 2016; Miki et al., 2018; Dakin et al., 2020; Markman et al., 2020) were included in this NMA (Supplementary Figure S1). Nine groups were included in the main network analysis: placebo (Pla), antidepressant (ADP), anti-NGF (ANGF), acetanilide antipyretic analgesics (AP), nonsteroidal anti-inflammatory drugs (NSAIDs), weak opioids (WO), strong opioids (SO), a combination of AP and WO (cAPWO), and a combination of NSAIDs and skeletal muscle relaxants (cNSMRs). The network plots of the main network analysis and subgroup analysis are presented in Figure 1 and Supplementary Figure S2, respectively.

### Study Characteristics

Nine thousand five hundred and eight patients were enrolled in this NMA. Across all studies, the mean age was 49.95 years (SD = 7.29), the median constituent ratio of male patients was 40.87% (interquartile = 44.29–49.62%), and the median length of follow-up was 12 weeks (interquartile distance = 2–13 weeks). Eight studies reported the relative treatment effects and safety for acute back pain (ABP), while only one study reported on sub-acute back pain (SABP) (Supplementary Table S1). The details of the studies’ quality and bias-risk assessments are shown in Supplementary Table S2.

Evidence contribution diagrams are presented in Supplementary Table S3–S6, and no obvious abnormalities were found. Funnel plots are presented in Supplementary Figure S3 and Supplementary Figure S4, and detailed results of the Egger’s tests are presented in Supplementary Figure S5 and Supplementary Figure S6.

### Efficacy Endpoints

Twenty-two studies with 8,699 patients and 25 studies with 7,072 patients were included in the pain relief and function improvement networks, respectively. As no inconsistencies were detected using either the global consistency test (Supplementary Figure S7) or node-split tests (Supplementary Figure S8), consistency models were used in both networks. No publication bias was found in either effect endpoint network.

Based on the SUCRA ranking, ANGF had the largest probability of being the best analgesic for LBP (SUCRA 82.1%), followed by cNSMRs (SUCRA 74.1%), and NSAIDs (SUCRA 61.7%), while ADP ranked the lowest (SUCRA 40.4%). However, only ANGF exhibited a significantly higher effect than Pla {SMD 0.89, 95% CI [(0.26, 1.51)]}.

Based on the SUCRA ranking, SOP had the highest probability of being the most effective treatment for improvement in function (SUCRA 83.6%), followed by ANGF (SUCRA 77.3%), and WOP (SUCRA 75.8%), while AP ranked the lowest (SUCRA 17.3%). Except for SOP {SMD 1.17, 95% CI [(0.13,2.21)]} and ANGF {SMD 0.93, 95% CI [(0.27,1.58)]}, no significant differences were observed among the other treatments and Pla.

### Safety Endpoints

Twenty-five studies with 8,537 patients were included in the TEAEs network. Significant inconsistency was detected with the global inconsistency test (*p* = 0.0001) and the node-split test (between ANGF and NSAIDs, as well as cAPWO and Pla). Next, a sensitivity analysis was conducted, then the sources of inconsistency and heterogeneity were excluded from the network (studies 9, 10, and 29). Subsequently, no inconsistency was reported in the reconstructed network, and the consistency model was adopted. No publication bias was detected. WOP [RR 1.15, 95% CI (1.00, 1.33); NNTH 14, 95% CI (7, 241)], SOP [RR 1.29, 95% CI (1.13, 1.46); NNTH 7, 95% CI (5, 17)], NSAIDs [RR 1.41, 95% CI (1.12, 1.77); NNTH 5, 95% CI (3, 18)], and cNSMRS [RR 2.02, 95% CI (1.35, 3.03); NNTH 2, 95% CI (1, 6)] all showed higher a incidence of TEAEs, while cNSMRs (SUCRA = 1.0%) ranked the lowest according to SUCRA.

Twenty-three studies with 8,517 patients were included in the SAEs network. As no inconsistency was detected, consistency models were used in this network. No publication bias was detected (Supplementary Figure S2 and Supplementary Figure S3). Eight treatments, except AP, were analyzed in the SAEs network. According to the SUCRA ranking, ANGF had the highest probability (SUCRA = 67.5%) to be the safest treatment, while ADP had the lowest probability (SUCRA = 16.1%). Only ADP exhibited a significantly higher risk of SAEs than Pla [RR 3.02, 95% CI (1.05, 8.62); NNTH 38, 95% CI (9, 1,543)].

**FIGURE 2:**
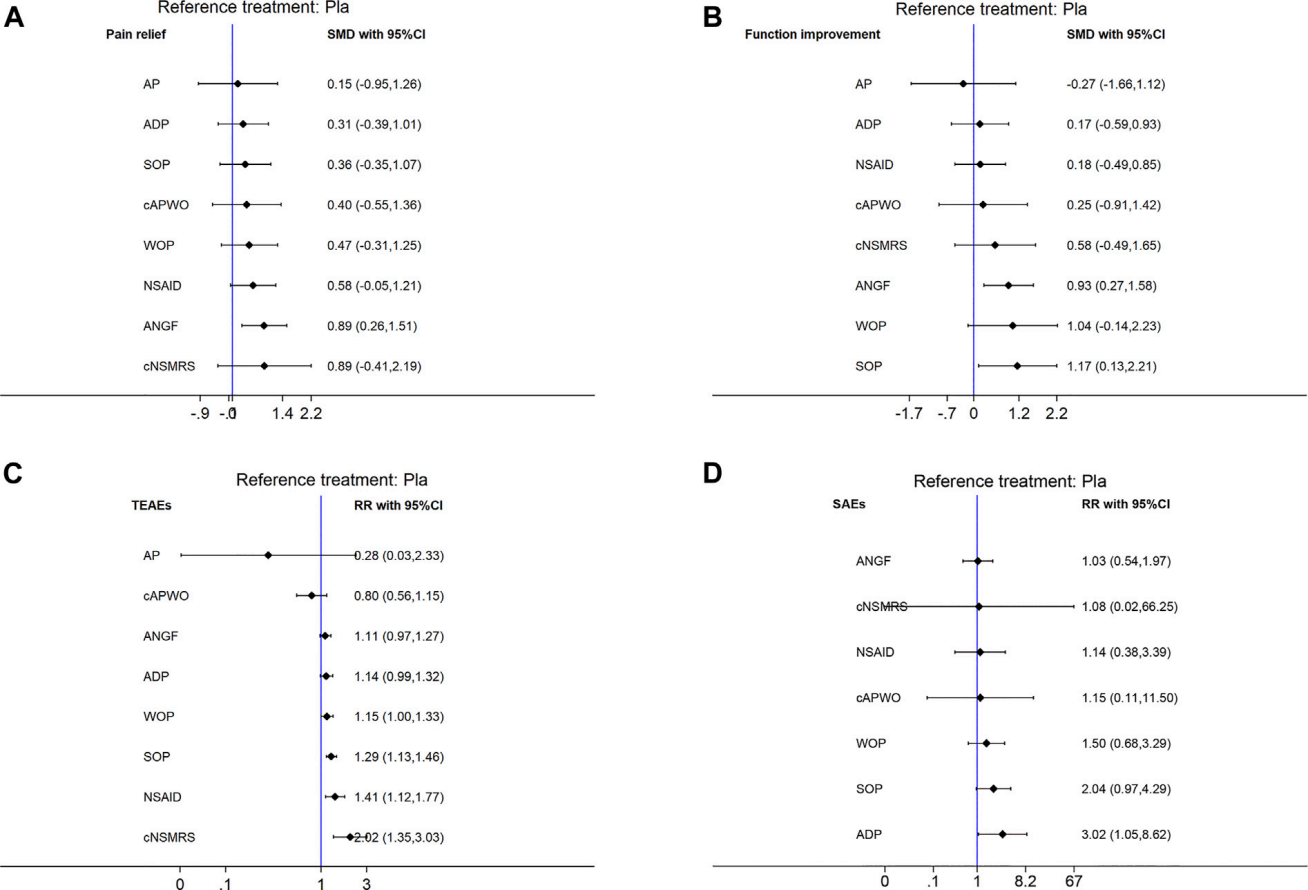
Forest plots of main analysis. SMD: Standardized mean differences; RR: risk ratio; CI: Confidence intervals. Differences between treatments were considered significant when the 95% CI did not contain 1 for RRs or 0 for SMDs. An intervention would be considered as a protective factor when its SMD less than 0 or RR less than 1, otherwise it would be risk factor. **(A)** Pain relief. **(B)** Function improvement. **(C)** TEAEs. **(D)** SAEs. Reference to Pla.

**FIGURE 3:**
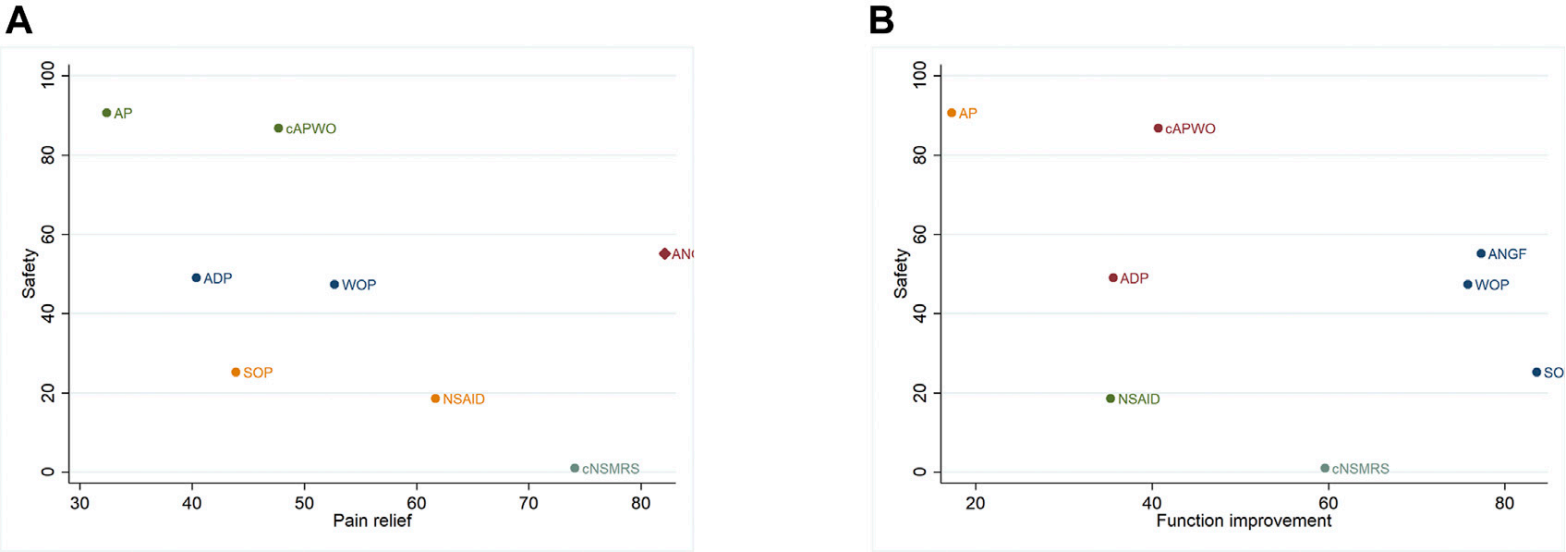
Cluset-rank plots of main analysis. Clustering for treatments of a network of interventions according to their performance on two outcomes, and the intervention closer to the upper right performed better. **(A)** The cluster-rank plot of pain relief and safety. **(B)** The cluster-rank plot of function improvement and safety (The cluster-rank value is the product of the abscissa and ordinate of each treatment).

Detailed SUCRA results are shown in Supplementary Figure S9 and Table 1. Forest plots are shown in Figure 2. The relative efficacy and safety between different treatments (league plots) are shown in Table 2 and Table 3. Based on the results of the cluster-rank (Figure 3), ANGF displayed the greatest potential to be the optimum treatment (cluster-rank value for function improvement and safety: 4266.96, and for pain relief and safety: 4531.92). NNTs plots were presented in Supplementary Figure S10.

**TABLE 1:** Detailed results of main analysis.

Treatment	SMD (95%CI) for pain relief	SURCA for pain relief, %	Mean rank for pain relief	SMD (95%CI) for function improvement	SURCA for function improvement, %	Mean rank for function improvement	RR (95%CI) for TEAEs	SURCA for TEAEs, %	Mean rank for TEAEs	RR (95%CI) for SAEs	SURCA for SAEs, %	Mean rank for SAEs
Pla	reference	14.9	7.8	reference	22.9	7.2	reference	76.4	2.9	reference	71.3	3.0
ADP	0.31 (−0.39,1.01)	40.4	5.8	0.17 (−0.59,0.93)	35.6	6.2	1.14 (0.99,1.32)	49.0	5.1	3.02 (1.05,8.62)	16.1	6.9
ANGF	0.89 (0.26,1.51)	82.1	2.4	0.93 (0.27,1.58)	77.3	2.8	1.11 (0.97,1.27)	55.2	4.6	1.03 (0.54,1.97)	67.5	3.3
AP	0.15 (−0.95,1.26)	32.4	6.4	−0.27 (−1.66,1.12)	17.3	7.6	0.28 (0.03,2.33)	90.6	1.8	NA	NA	NA
cAPWO	0.40 (−0.55,1.36)	47.7	5.2	0.25 (−0.91,1.42)	40.7	5.7	0.80 (0.56,1.15)	86.8	2.1	1.15 (0.11,11.50)	56.0	4.1
NSAID	0.58 (−0.05,1.21)	61.7	4.1	0.18 (−0.49,0.85)	35.3	6.2	1.41 (1.12,1.77)	18.6	7.5	1.14 (0.38,3.39)	60.0	3.8
cNSMRS	0.89 (−0.41,2.19)	74.1	3.1	0.58 (−0.49,1.65)	59.6	4.2	2.02 (1.35,3.03)	1.0	8.9	1.08 (0.02,66.25)	55.3	4.1
SOP	0.36 (−0.35,1.07)	43.9	5.5	1.17 (0.13,2.21)	83.6	2.3	1.29 (1.13,1.46)	25.2	7.0	2.04 (0.97,4.29)	28.0	6.0
WOP	0.47 (−0.31,1.25)	52.7	4.8	1.04 (−0.14,2.23)	75.8	2.8	1.15 (1.00,1.33)	47.3	5.2	1.50 (0.68,3.29)	45.8	4.8

**TABLE 2:** The league plots of main efficacy analysis. Pain relief (Red) and function improvement (Blue). (From the top left to the bottom right, higher comparator vs lower comparator, SMD with 95% CI.)

ANGF	−0.35 (−1.53, 0.83)	−0.75 (−1.59, 0.08)	0.11 (−1.13, 1.35)	−0.67 (−2.01, 0.66)	−0.76 (−1.74, 0.23)	0.24 (−0.95, 1.43)	−1.20 (−2.68, 0.28)	−0.93 (−1.58, −0.27)
−0.01 (−1.39, 1.38)	cNSMRS	−0.40 (−1.23, 0.43)	0.46 (−1.11, 2.04)	−0.32 (−1.90, 1.26)	−0.41 (−1.65, 0.84)	0.59 (−0.89, 2.07)	−0.85 (−2.32, 0.62)	−0.58 (−1.65, 0.49)
0.31 (−0.48, 1.10)	0.32 (−0.82, 1.45)	NSAID	0.86 (−0.48, 2.20)	0.08 (−1.27, 1.42)	−0.01 −0.93, 0.92)	0.99 (−0.24, 2.22)	-0.45 (−1.66, 0.77)	−0.18 (−0.85, 0.49)
0.41 (−0.52, 1.35)	0.42 (−1.09, 1.93)	0.10 (−0.89, 1.09)	WOP	−0.79 (−2.45, 0.88)	−0.87 (−2.27, 0.53)	0.13 (−1.11, 1.37)	−1.31 (−3.12, 0.50)	−1.04 (−2.23, 0.14)
0.48 (−0.64, 1.61)	0.49 (−1.12, 2.10)	0.17 (−0.97, 1.31)	0.07 (−0.99, 1.13)	cAPWO	−0.08 (−1.48, 1.31)	0.91 (−0.65, 2.48)	−0.53 (−2.34, 1.29)	−0.25 (−1.42, 0.91)
0.58 (−0.34, 1.50)	0.59 (−0.82, 1.99)	0.27 (−0.55, 1.09)	0.17 (−0.88, 1.21)	0.10 (−1.09, 1.28)	ADP	1.00 (−0.29, 2.28)	−0.44 (−1.97, 1.09)	−0.17 (−0.93, 0.59)
0.53 (−0.39, 1.45)	0.53 (−0.95, 2.01)	0.22 (−0.73, 1.16)	0.11 (−0.66, 0.89)	0.04 (−1.08,1.16)	−0.05 (−1.05, 0.94)	SOP	−1.44 (−3.17, 0.29)	−1.17 (−2.21, −0.13)
0.73 (−0.49, 1.95)	0.74 (−0.78, 2.26)	0.42 (−0.58, 1.43)	0.32 (−1.02, 1.66)	0.25 (−1.21, 1.71)	0.15 (−0.96, 1.27)	0.21 (−1.10, 1.51)	AP	0.27 (−1.12, 1.66)
0.89 (0.26, 1.51)	0.89 (−0.41, 2.19)	0.58 (−0.05, 1.21)	0.47 (−0.31, 1.25)	0.40 (−0.55, 1.36)	0.31 (−0.39, 1.01)	0.36 (−0.35, 1.07)	0.15 (−0.95, 1.26)	Pla

**TABLE 3:** The league plots of main safety analysis. TEAEs (Red) and SAEs (Blue). (From the top left to the bottom right, higher comparator vs lower comparator, RR with 95% CI.)

ANGF	—	1.11 (0.10,11.96)	1.45 (0.60,3.52)	2.92 (0.85,10.08)	1.97 (0.79,4.95)	1.10 (0.35,3.50)	1.05 (0.02,65.30)	0.97 (0.51,1.85)
3.95 (0.48, 32.82)	AP	—	—	—	—	—	—	—
1.39 (0.95, 2.02)	0.35 (0.04, 3.01)	cAPWO	1.31 (0.12, 13.70)	2.63 (0.21, 32.99)	1.78 (0.16, 19.25)	0.99 (0.08, 12.63)	0.94 (0.01, 105.11)	0.87 (0.09, 8.75)
0.96 (0.81, 1.14)	0.24 (0.03, 2.03)	0.69 (0.50, 0.97)	WOP	2.01 (0.56, 7.25)	1.36 (0.63, 2.92)	0.76 (0.21, 2.79)	0.72 (0.01, 46.97)	0.67 (0.30, 1.46)
0.97 (0.80, 1.18)	0.25 (0.03, 2.05)	0.70 (0.47, 1.04)	1.01 (0.82, 1.24)	ADP	0.68 (0.19, 2.43)	0.38 (0.08, 1.69)	0.36 (0.01, 24.93)	0.33 (0.12, 0.95)
0.86 (0.73, 1.02)	0.22 (0.03, 1.82)	0.62 (0.44, 0.89)	0.90 (0.80, 1.01)	0.89 (0.73, 1.08)	SOP	0.56 (0.15, 2.06)	0.53 (0.01, 34.58)	0.49 (0.23, 1.04)
0.79 (0.61, 1.03)	0.20 (0.02, 1.64)	0.57 (0.37, 0.88)	0.82 (0.62, 1.09)	0.81 (0.62, 1.06)	0.91 (0.70, 1.20)	NSAID	0.95 (0.02, 50.25)	0.88 (0.29, 2.62)
0.55 (0.36, 0.84)	0.14 (0.02, 1.17)	0.40 (0.23, 0.68)	0.57 (0.37, 0.88)	0.56 (0.37, 0.87)	0.64 (0.41, 0.97)	0.70 (0.50, 0.97)	cNSMRS	0.92 (0.02, 56.61)
1.11 (0.97, 1.27)	0.28 (0.03, 2.33)	0.80 (0.56, 1.15)	1.15 (1.00, 1.33)	1.14 (0.99, 1.32)	1.29 (1.13, 1.46)	1.41 (1.12, 1.77)	2.02 (1.35, 3.03)	Pla

### Subgroup Analyses

Two subgroup analyses were performed. After excluding nine studies that focused on ALBP or SABP, 21 trials with 7,751 patients were included in the first subgroup analysis. Eight drugs, except cNSMRs, were included in the subgroup efficacy networks. No inconsistency was reported, so a consistency random-effects model was built. ANGF showed a superior effect than Pla for pain relief [SUCRA 86.9%, SMD 1.04, 95% CI (0.29,1.79)], and for improvement in function [SUCRA 76.1%, SMD 0.99, 95% CI (0.18,1.80)]. A significant difference was no longer observed for improved function between SO and Pla for CLBP.

Seven drugs, except cNSMRs and AP, were included in the subgroup safety networks. Significant inconsistency was detected in the sub-TEAEs network. This unexplained inconsistency was not eliminated, nor could the source be identified by additional sensitivity analysis. Therefore, an inconsistency model was adopted in the sub-TEAEs network, and a consistency model was adopted in the sub-SAEs network. Apart from WOP [RR 1.52, 95%CI (1.15,2.01)], SOP [RR 1.48, 95%CI (1.20,1.83)], and ADP [RR 1.14, 95%CI (1.01,1.29)], other treatments did not have more TEAEs or SAEs than Pla. ADP also showed a higher incidence of SAEs than Pla [RR 3.16, 95% CI (1.02,9.72)]. Consistent with the results of the main network analysis, only ANGF exhibited a significant efficacy on CLBP. Detailed results and league plots are presented in Supplementary Figure S11 and Supplementary Table S7, Supplementary Table S8.

In the second subgroup analysis, five studies involving three ANGFs (fasinumab, tanezumab, and fulranumab) were analyzed. According to the SUCRA ranking, tanezumab had the highest probability of being the most effective ANGF for pain relief (SUCRA = 76.7%) and function improvement (SUCRA = 73.7%). In comparison, fasinumab had the highest probability of being the safest ANGF (SUCRA = 60.8% for TEAEs network and 53.5% for SAEs network). However, the differences were not statistically significant (Supplementary Figure S12 and Supplementary Table S9, Supplementary Table S10). However, it is worth noting that one study (Skljarevski et al., 2010b) reported a special risk of adjudicated arthropathies (AAs). Nineteen arthropathies were observed in 16 patients who received fasinumab treatment, and no primary osteonecrosis was observed. Most of the reported AAs were rapidly progressing osteoarthritis, and 18 of the 19 occurred in patients who already exhibited peripheral osteoarthritis (screening K-L scores of ≥2 at the knee and hip, or screening radiographs indicated moderate to severe osteoarthritis) at the baseline evaluation. Eventually, two patients underwent joint replacement surgery due to AAs.

## Discussion

LBP is a common disease but without a clear etiology and pathological process. Although non-pharmacologic treatments have been recommended as the initial choice for LBP treatment based on clinical guidelines, pharmacologic treatments often are still necessary, especially for patients who do not respond adequately to non-pharmacologic therapy. However, traditional drugs, including NSAIDs, tramadol, and duloxetine, only provide a small or even no improvement in pain and function. Thus, the emergence of targeting inhibition of nerve growth factor clearly provides new possibilities and directions for LBP treatment. Numerous systematic reviews and meta-analysis studies have already been conducted to assess pharmacologic treatments for LBP. A study by Ferreira et al. (2021) provides moderate evidence that SSRIs, NDRIs, and SARIs in antidepressants had little effect on pain and disability scores and had no clinical significance for the relief of back pain. These observations are consistent with our results that ADP was not suitable for LBP treatment due to a lack of effect and the risk of SAEs.

Leite et al. (2014) found anti-NGF, specifically, tanezumab exhibited a low-to-moderate effect on pain relief in LBP with relatively lower evidence of adverse effects compared to placebo. The analysis by Yang et al. (2020) focused on three types of anti-NGF antibodies and found they could relieve pain and improve function in patients with osteoarthritis pain and LBP. However, these results lacked comparisons among the ANGFs with other pharmacological treatments for LBP due to the study design and the limited number of studies that were included. Therefore, these studies did not provide enough information to help make reliable clinical decisions.

Based on these previous meta-analyses, we conducted the first NMA to synthesize all relevant, high-quality RCTs to comprehensively compare the effects and safety of ANGFs with other guideline-recommended drugs in the treatment of LBP. The main findings are as follows. 1). ANGFs were associated with a significant decrease in pain intensity, while NSAIDs, opioids, SMRs, and ADPs exhibited only a small or no decrease in LBP pain compared with placebo. 2). ANGFs and SO exhibited a significant effect on improving function in the main analysis. However, after excluding all studies limited to ALBP or SABP, SO no longer showed a better effect than placebo. This result implied that SO could only bring about significant function improvement for ALBP or SALBP rather than CLBP. 3). According to the results of the TEAEs and SAEs networks, ANGFs were well-tolerated and safe for the treatment of LBP. However, opioids, NSAIDs, and SMRs exhibited a lower tolerance than placebo, and ADPs demonstrated a higher risk for SAE than placebo. 4). No significant difference was observed between the efficacy and safety of the three ANGFs.

There are some limitations to this study. First, only studies using a parallel, randomized, controlled design were included to reduce the influence of possible confounding factors on the results. This decision led to the inclusion of only a small number of studies in the analysis. Although the funnel plot and Egger’s test did not reveal any significant results, publication bias and small-study effects still could have been potential problems in such a sparse network. Second, the median follow-up period was 12 weeks, which was relatively short. It is challenging to accurately assess long-term safety profiles, especially for AEs that occur with moderate to low incidence in such a relatively short time compared to studies with a longer follow-up period. This relatively short follow-up time might also be due to the exclusion of observational studies. Third, SUCRA is commonly used to rank the relative effects and choose optimal treatments (Zeng et al., 2018). However, the results of SUCRA should be used with caution because the differences between treatments might be insignificant in clinical settings. Although the results were assessed using SUCRA, the absolute difference between the highest ranked therapy and the others could be minimal (Wang and Carter, 2018). Last, it could not be ignored that ANGFs appeared to be associated with an increased risk of AAs. It is possible that ANGFs could lead to a rapid progression of arthropathy on the basis of osteoarthritis and even require arthroplasty treatment. The Food and Drug Administration (FDA) has placed ANGFs studies on hold for this reason (Research. USFaDACfDEa, 2012a; Research. USFaDACfDEa, 2012b). The use of ANGFs for arthropathy should be considered cautiously, especially for patients who already have peripheral osteoarthritis. However, another systematic review (Leite et al., 2014) reported that only one case of AAs occurred out of 1,325 patients receiving ANGFs for LBP. This result is consistent with our findings that most of the recorded AAs occurred relative to peripheral osteoarthritis. The use of ANGFs to treat LBP seems to be safer than for OA. Although the results suggest that ANGF have the potential to become a new first-line drug for the treatment of LBP, more studies are still needed to confirm the safety of ANGF in consideration of its serious joint toxicity.

## Conclusion

This NMA identified 30 studies involving 9,508 patients with LBP. ANGFs relieved pain and improved function effectively and were superior to other traditional drugs recommended by published guidelines. Although no significant differences in tolerability and safety between ANGFs and placebo were observed, the rapid progression of original osteoarthritis, which might be related to the use of ANGFs, needs specific attention and additional verification through clinical trials.

## Data Availability

The raw data supporting the conclusion of this article will be made available by the authors, without undue reservation.
