# Supplementary material for: Is Targeting Nerve Growth Factor Antagonist a New Option for Pharmacologic Treatment of Low Back Pain? A Supplemental Network Meta-Analysis of the American College of Physicians Guidelines
Source: Front Pharmacol. 2021 Aug 31;12:727771. doi: 10.3389/fphar.2021.727771 (PMC8438173; doi:10.3389/fphar.2021.727771)
Supplement: Supplementary file 2 [file DataSheet1.docx]

**Supplemental figure 1.** PRISMA Flow Diagram.

**Supplemental figure 2.** Structure of subgroup network formed by interventions. The lines between treatment nodes indicate the direct comparisons made within randomised controlled trials. (1) Pain relief. (2) Function improvement. (3) TEAEs. (4) SAEs. (A: Pla; B: ADP; C: ANGF; D: AP; E: cAPWO; F: NSAID; G: SOP; H: WOP.)

**Supplemental figure 3.** Funnel plots for main analysis. (1) Pain relief. (2) Function improvement. (3) TEAEs. (4) SAEs. (A: Pla; B: ADP; C: ANGF; D: AP; E: cAPWO; F: NSAID; G: cNSMRS; H: SOP; I: WOP.)

**Supplemental figure 4.** Funnel plots for subgroup analysis. (1) Pain relief. (2) Function improvement. (3) TEAEs. (4) SAEs. (A: Pla; B: ADP; C: ANGF; D: AP; E: cAPWO; F: NSAID; G: SOP; H: WOP.)

**Supplemental figure 5.** Egger’s tests for main analysis. (1) Pain relief. (2) Function improvement. (3) TEAEs. (4) SAEs.

**Supplemental figure 6.** Egger’s tests for subgroup analysis. (1) Pain relief. (2) Function improvement. (3) TEAEs. (4) SAEs.

**Supplemental figure 7.** Global consistency tests and synthesized forest plots for main analysis. (1) Pain relief. (2) Function improvement. (3) TEAEs. (4) SAEs.

**Supplemental figure 8.** Global consistency tests and synthesized forest plots for main analysis. (1) Pain relief. (2) Function improvement. (3) TEAEs. (4) SAEs.

**Supplemental figure 9.** Detailed SUCRA ranking for main analysis. (1) Pain relief. (2) Function improvement. (3) TEAEs. (4) SAEs.

**Supplemental figure 10. NNTs plot for the safety outcomes of** main analysis. (1) TEAEs. (2) SAEs.(NNTs: Numbers needed to treat; NNTB: Number needed to treat for an additional beneficial outcome; NNTH: number needed to treat for an additional harmful outcome.)

**Supplemental figure 11.** Forest plots of subgroup analysis. (1) Pain relief. (2) Function improvement. (3) TEAEs. (4) SAEs. Reference to Pla. (SMD: Standardized mean differences; RR: risk ratio; CI: Confidence intervals.)

**Supplemental figure 12.** Forest plots of the second subgroup analysis. (1) Pain relief. (2) Function improvement. (3) TEAEs. (4) SAEs. Reference to Placebo. (SMD: Standardized mean differences; RR: risk ratio; CI: Confidence intervals.)

**Supplemental table 1.** Baseline Characteristics of included Studies.

**Supplemental table 2.** Methodological quality and risk of bias evaluation of included study. L: low risk of bias.

**Supplemental table 3**. Evidence contribution diagram for the pain relief network. The numbers are to the weight attached to each direct summary effect (horizontal axis) for the estimation of each network summary effects (vertical axis) as percentages. (A: Pla; B: ADP; C: ANGF; D: AP; E: cAPWO; F: NSAID; G: cNSMRS; H: SOP; I: WOP.)

**Supplemental table 4**. Evidence contribution diagram for the function improvement network. The numbers are to the weight attached to each direct summary effect (horizontal axis) for the estimation of each network summary effects (vertical axis) as percentages. (A: Pla; B: ADP; C: ANGF; D: AP; E: cAPWO; F: NSAID; G: cNSMRS; H: SOP; I: WOP.)

**Supplemental table 5**. Evidence contribution diagram for the TEAEs network. The numbers are to the weight attached to each direct summary effect (horizontal axis) for the estimation of each network summary effects (vertical axis) as percentages. (A: Pla; B: ADP; C: ANGF; D: AP; E: cAPWO; F: NSAID; G: cNSMRS; H: SOP; I: WOP.)

**Supplemental table 6**. Evidence contribution diagram for the SAEs network. The numbers are to the weight attached to each direct summary effect (horizontal axis) for the estimation of each network summary effects (vertical axis) as percentages. (A: Pla; B: ADP; C: ANGF; D: cAPWO; E: NSAID; F: cNSMRS; G: SOP; H: WOP.)

**Supplemental table 7**. The league plots of subgroup efficacy analysis. Pain relief (Red) and function improvement (Blue). (From the top left to the bottom right, higher comparator vs lower comparator, SMD with 95% CI.)

**Supplemental table 8**. The league plots of subgroup safety analysis. TEAEs (Red) and SAEs (Blue). (From the top left to the bottom right, higher comparator vs lower comparator, RR with 95% CI.)

**Supplemental table 9**. The league plots of the second subgroup efficacy analysis. Pain relief (Red) and function improvement (Blue). (From the top left to the bottom right, higher comparator vs lower comparator, SMD with 95% CI.)

**Supplemental table 10**. The league plots of the second subgroup safety analysis. TEAEs (Red) and SAEs (Blue). (From the top left to the bottom right, higher comparator vs lower comparator, RR with 95% CI.)


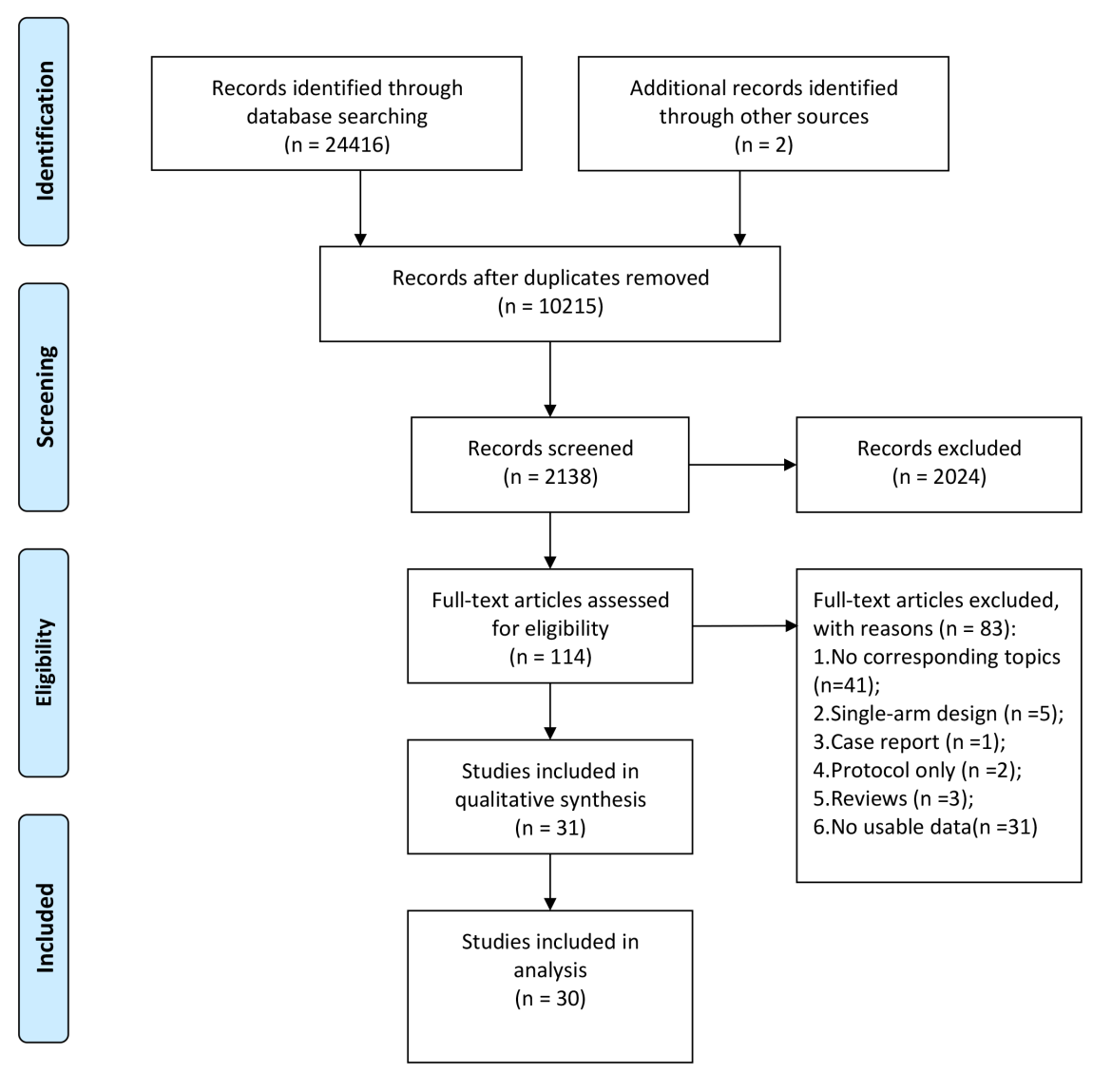


**Supplemental figure 1.** PRISMA Flow Diagram.

**
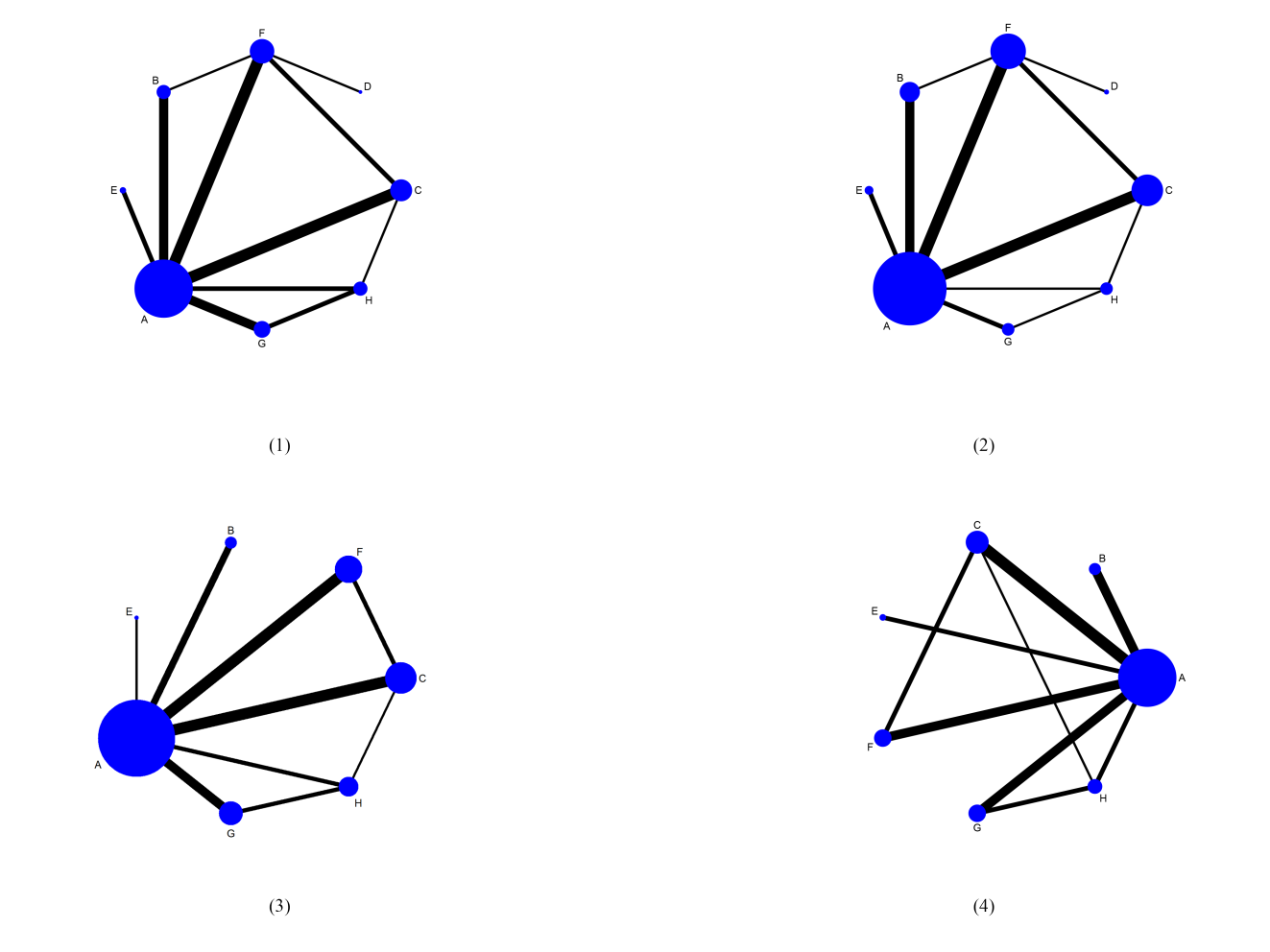
**

**Supplemental figure 2.** Structure of subgroup network formed by interventions. The lines between treatment nodes indicate the direct comparisons made within randomised controlled trials. (1) Pain relief. (2) Function improvement. (3) TEAEs. (4) SAEs. (A: Pla; B: ADP; C: ANGF; D: AP; E: cAPWO; F: NSAID; G: SOP; H: WOP.)


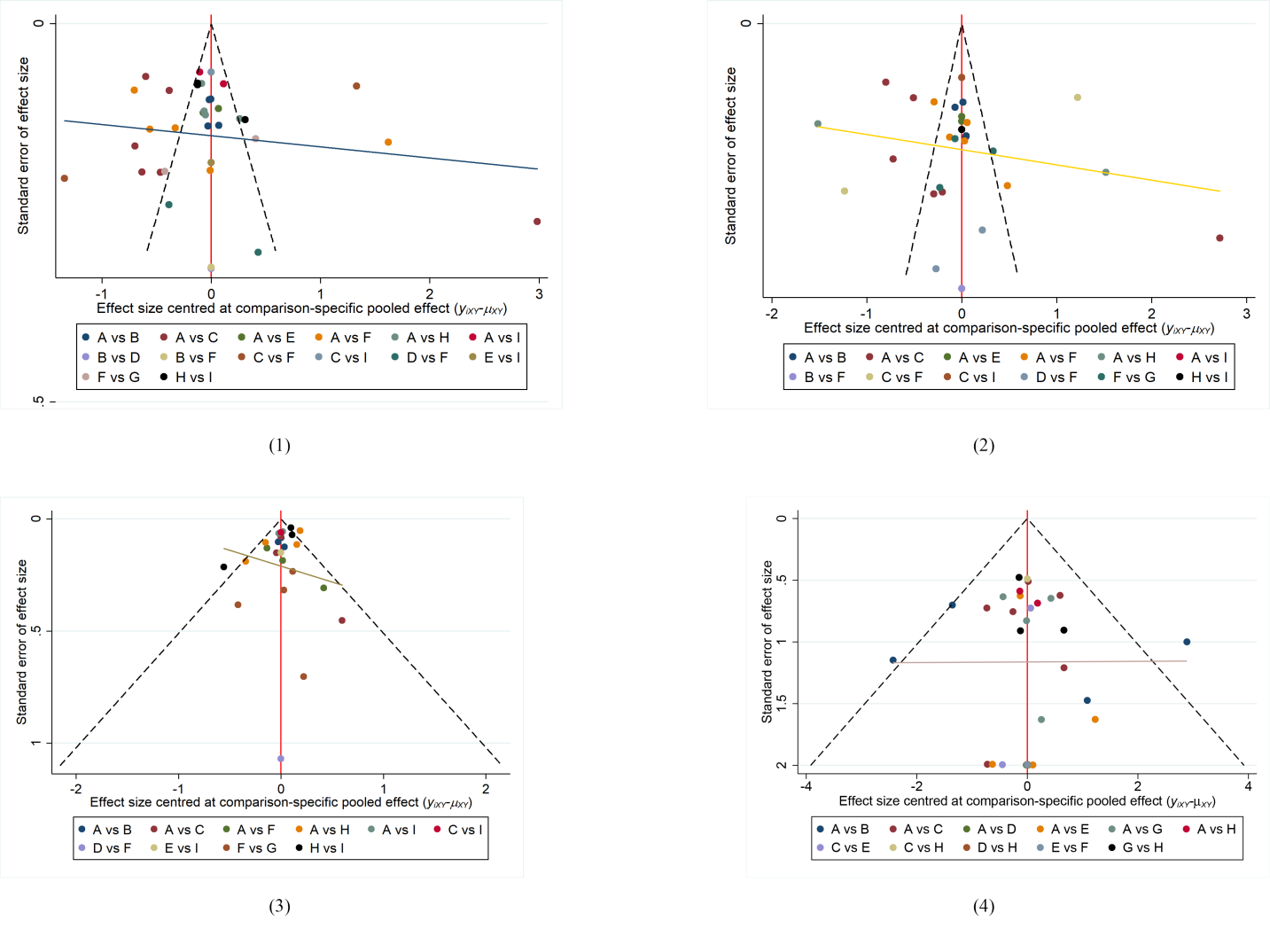


**Supplemental figure 3.** Funnel plots for main analysis. (1) Pain relief. (2) Function improvement. (3) TEAEs. (4) SAEs. (A: Pla; B: ADP; C: ANGF; D: AP; E: cAPWO; F: NSAID; G: cNSMRS; H: SOP; I: WOP.)


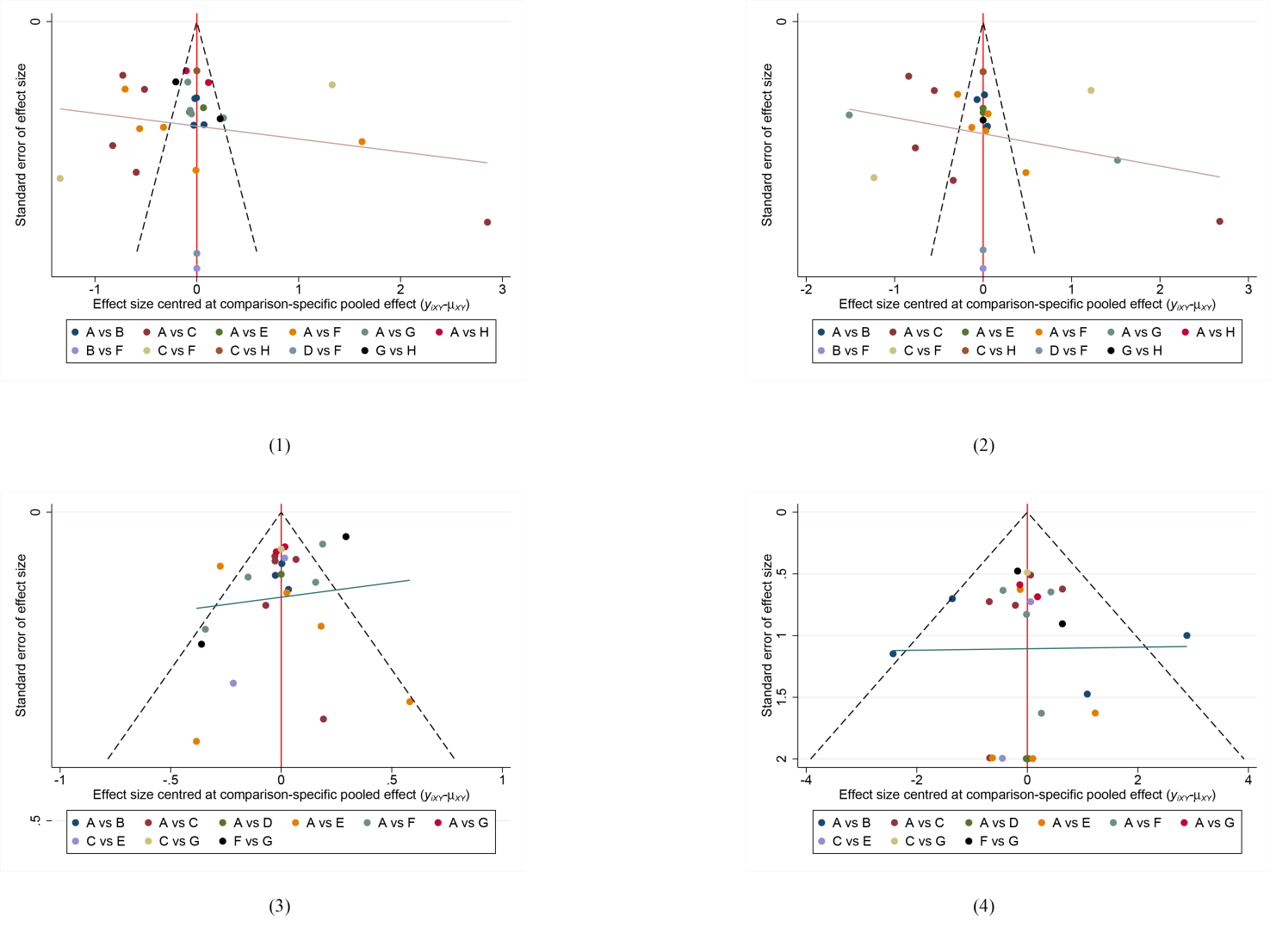


**Supplemental figure 4.** Funnel plots for subgroup analysis. (1) Pain relief. (2) Function improvement. (3) TEAEs. (4) SAEs. (A: Pla; B: ADP; C: ANGF; D: AP; E: cAPWO; F: NSAID; G: SOP; H: WOP.)


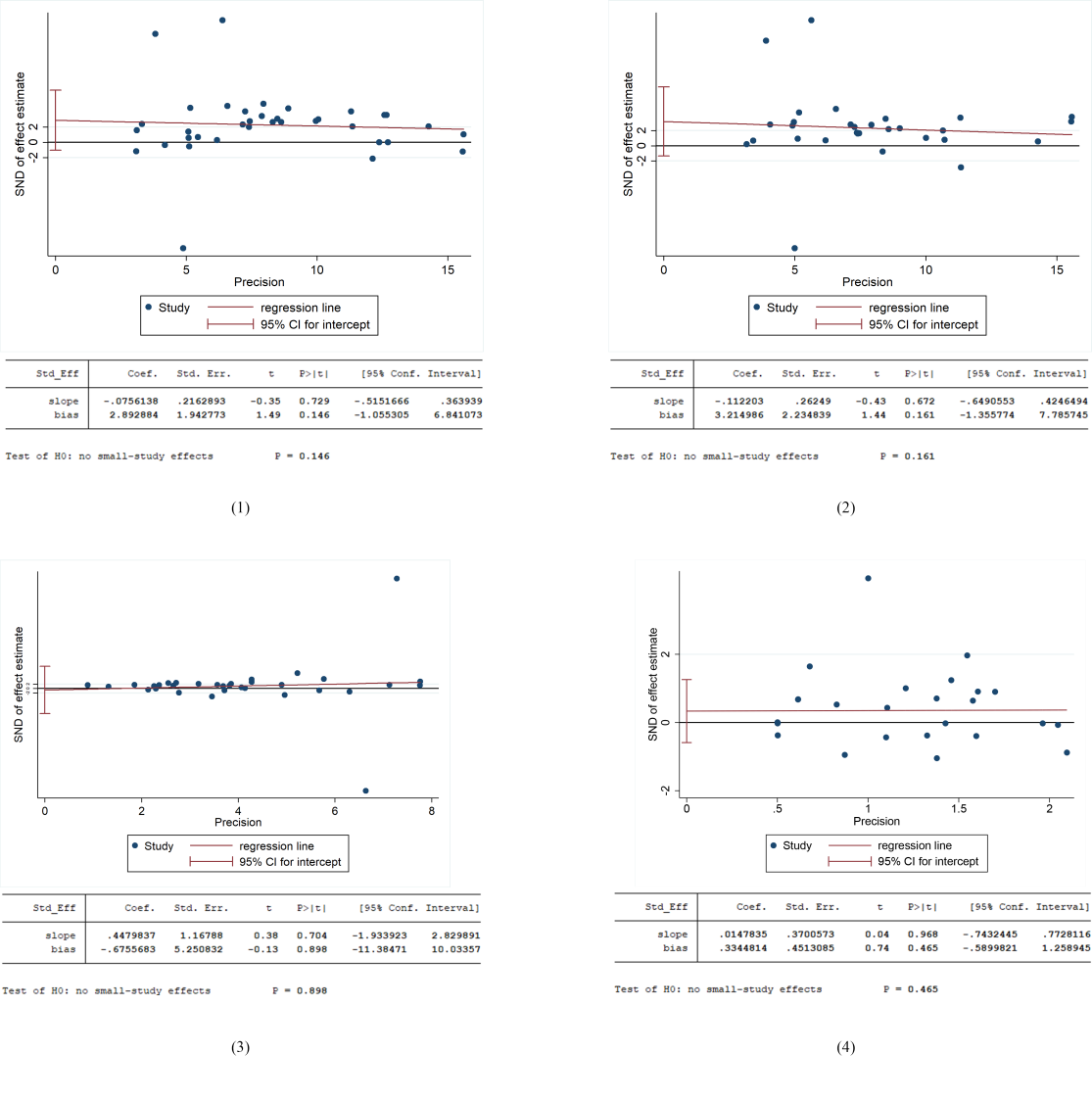


**Supplemental figure 5.** Egger’s tests for main analysis. (1) Pain relief. (2) Function improvement. (3) TEAEs. (4) SAEs.


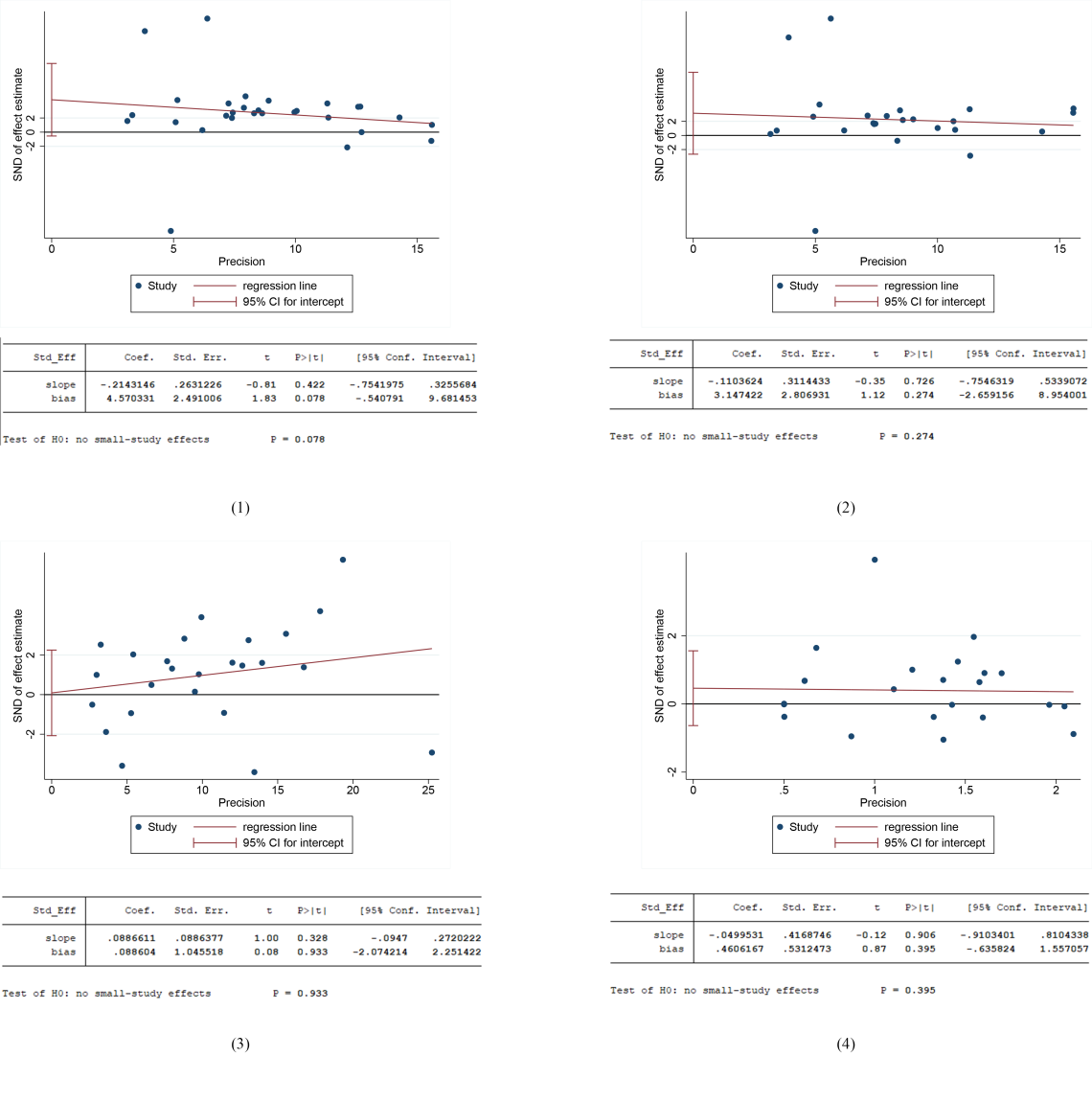


**Supplemental figure 6.** Egger’s tests for subgroup analysis. (1) Pain relief. (2) Function improvement. (3) TEAEs. (4) SAEs.


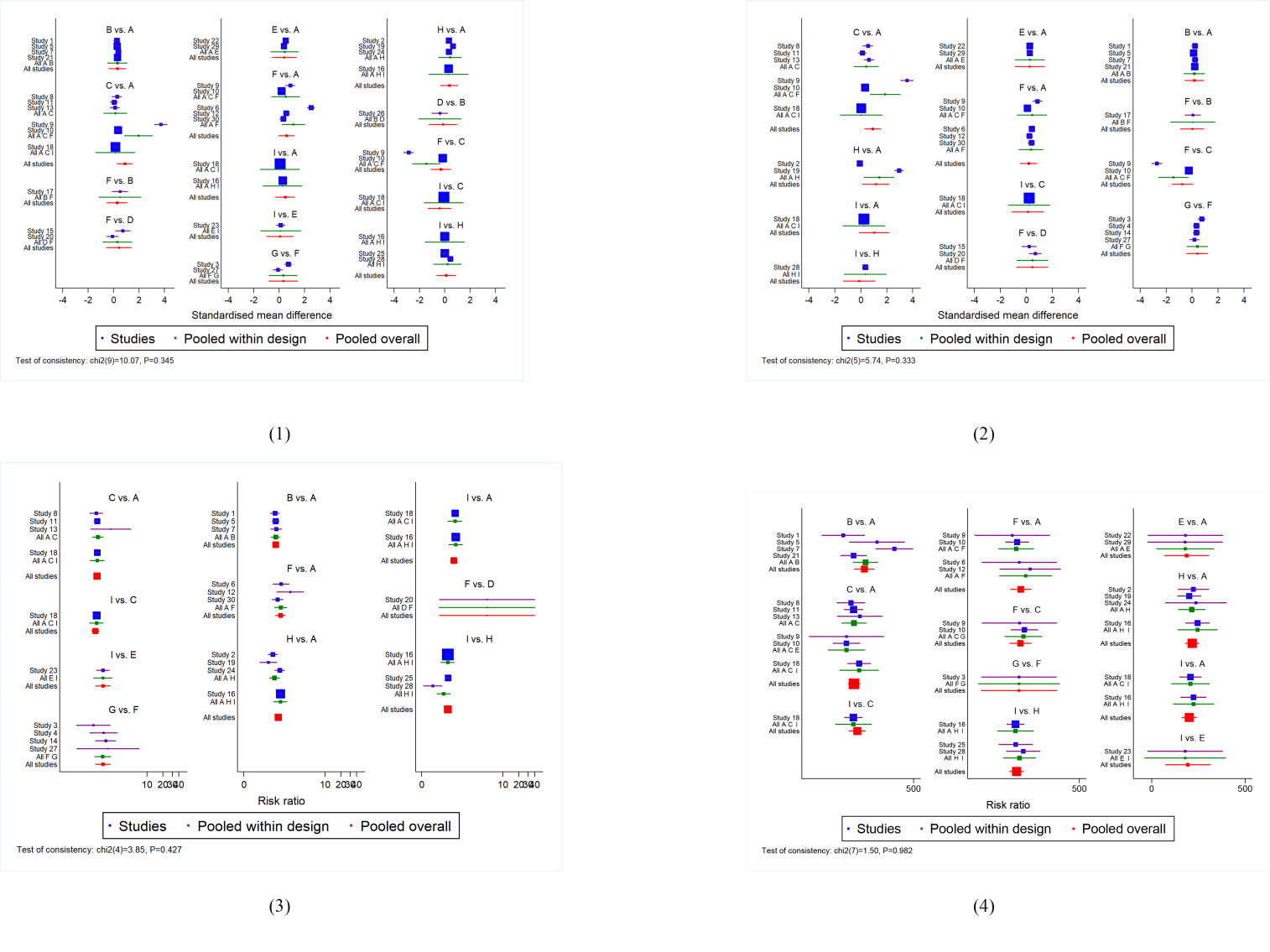


**Supplemental figure 7.** Global consistency tests and synthesized forest plots for main analysis. (1) Pain relief. (2) Function improvement. (3) TEAEs. (4) SAEs.

**
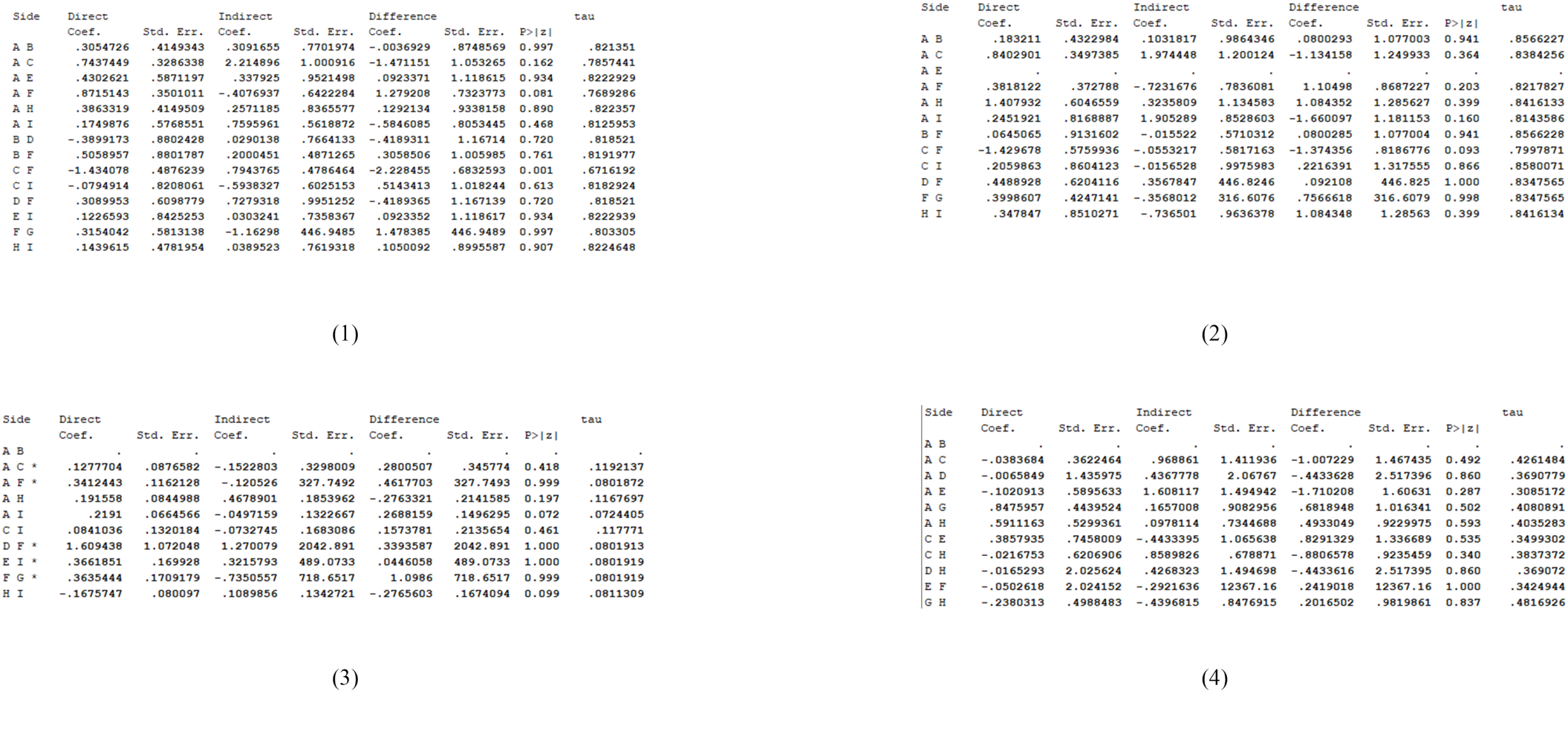
**

**Supplemental figure 8.** Global consistency tests and synthesized forest plots for main analysis. (1) Pain relief. (2) Function improvement. (3) TEAEs. (4) SAEs.


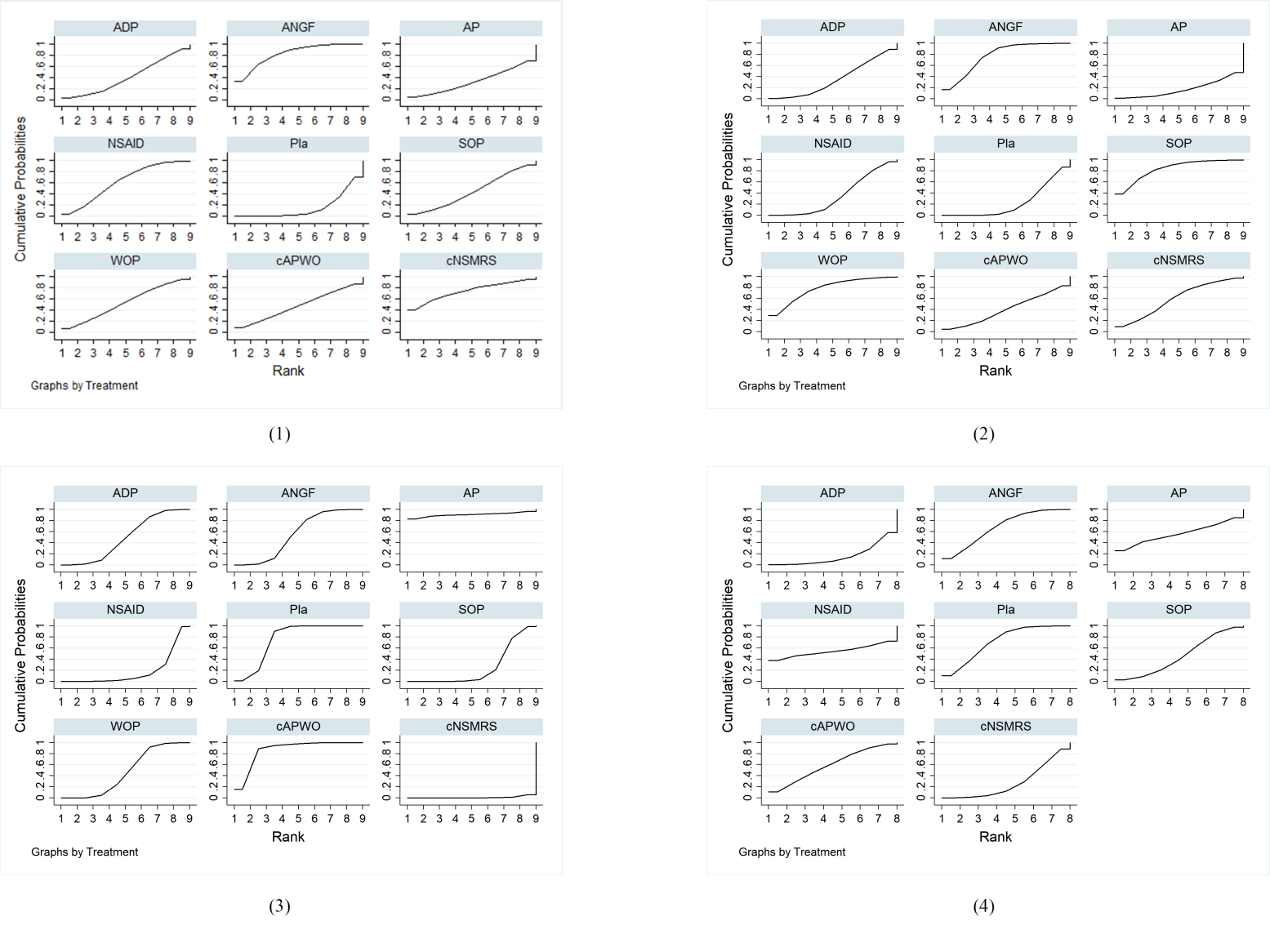


**Supplemental figure 9.** Detailed SUCRA ranking for main analysis. (1) Pain relief. (2) Function improvement. (3) TEAEs. (4) SAEs.


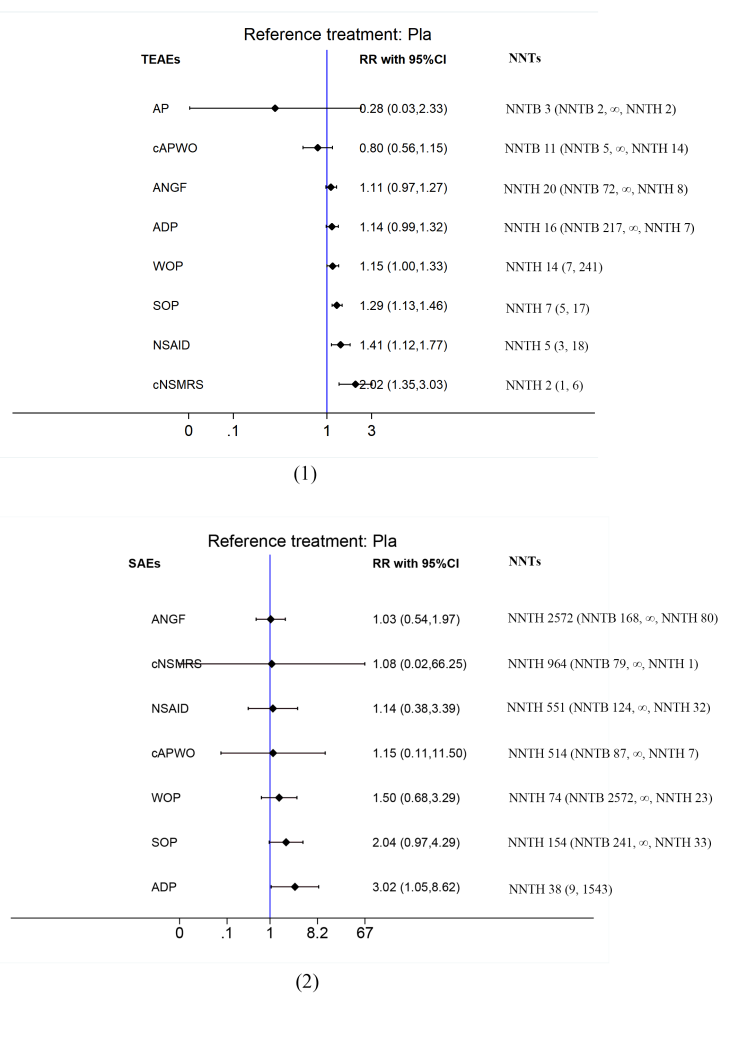


**Supplemental figure 10. NNTs plot for the safety outcomes of** main analysis. (1) TEAEs. (2) SAEs.(NNTs: Numbers needed to treat; NNTB: Number needed to treat for an additional beneficial outcome; NNTH: number needed to treat for an additional harmful outcome.)

**
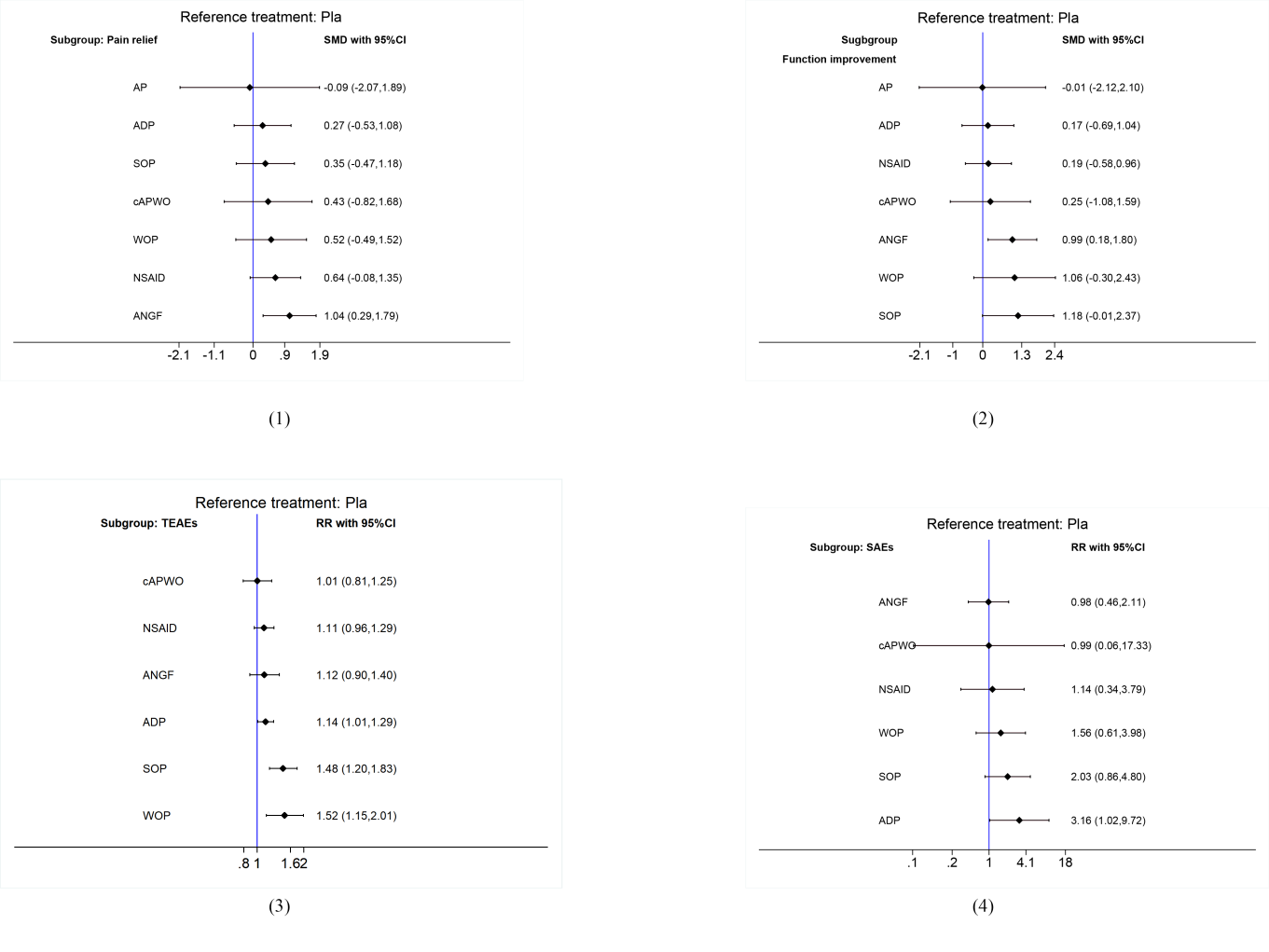
**

**Supplemental figure 11.** Forest plots of subgroup analysis. (1) Pain relief. (2) Function improvement. (3) TEAEs. (4) SAEs. Reference to Pla. (SMD: Standardized mean differences; RR: risk ratio; CI: Confidence intervals.)


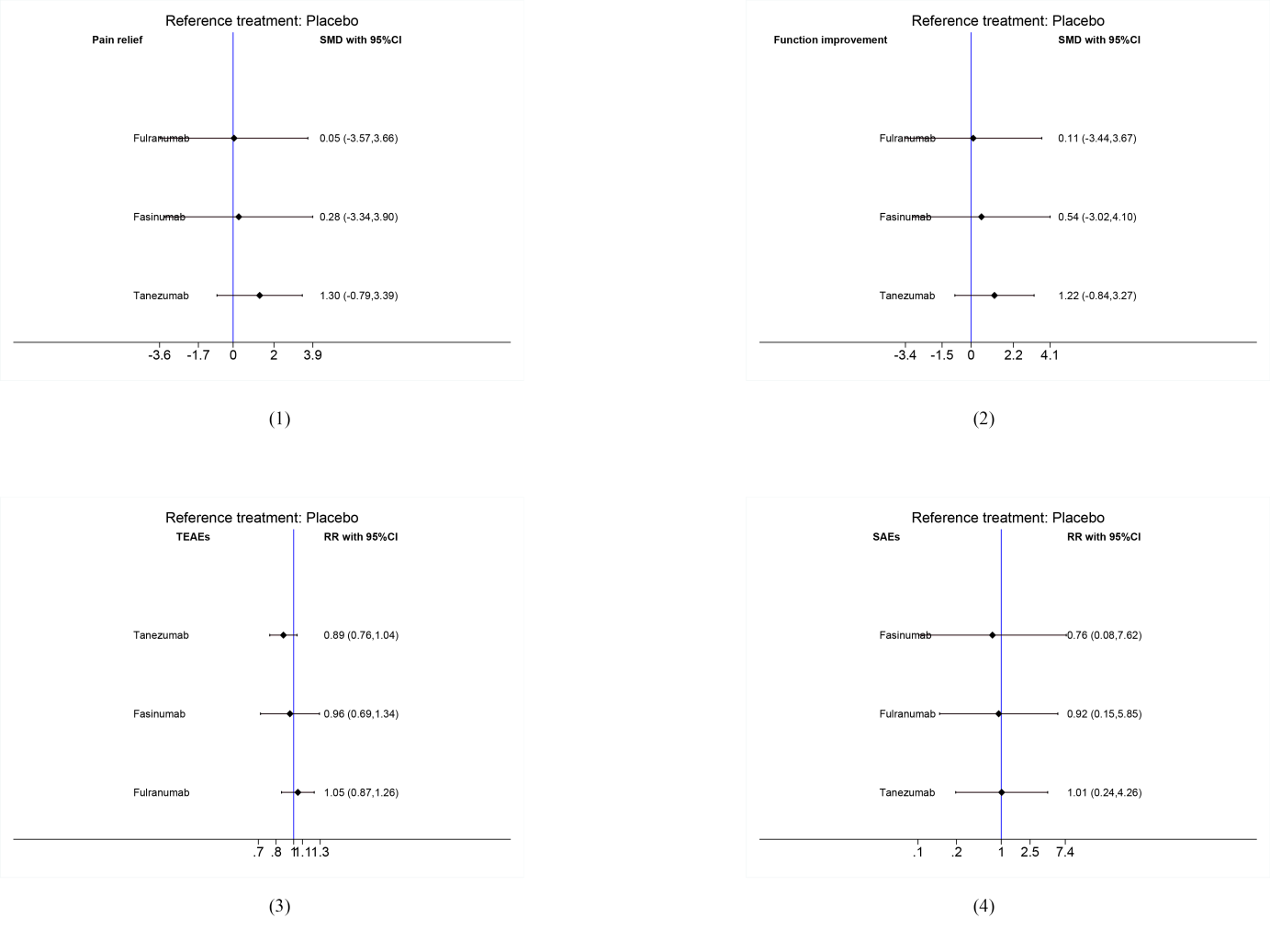


**Supplemental figure 12.** Forest plots of the second subgroup analysis. (1) Pain relief. (2) Function improvement. (3) TEAEs. (4) SAEs. Reference to Placebo. (SMD: Standardized mean differences; RR: risk ratio; CI: Confidence intervals.)

**Supplemental table 1.** Baseline Characteristics of included Studies

| Author | No. | Year | Number of  patients | Mean age | Percentage of Male (%) | Disease type | Mean follow-up period (weeks) | Intervention I | Intervention II | Intervention III |
| --- | --- | --- | --- | --- | --- | --- | --- | --- | --- | --- |
| Skljarevski V et. al. | 1 | 2009 | 233 | 53.65 | 43.78 | CLBP | 13 | Placebo | Duloxetine | NA |
| Rauck RL et. al. | 2 | 2015 | 280 | 49.98 | 44.29 | CLBP | 2 | Placebo | oxycodone | NA |
| Pareek A et. al. | 3 | 2009 | 197 | 43.32 | 60.91 | ALBP | 1 | Aceclofenac combined with Tizanidine | Aceclofenac | NA |
| Gottlieb M et. al. | 4 | 2016 | 215 | NR | NR | ALBP | 1 | Naproxen combined with cyclobenzaprine | Naproxen | NA |
| Skljarevski V et. al. | 5 | 2010 | 401 | 54.14 | 38.65 | CLBP | 12 | Placebo | Duloxetine | NA |
| Coats TL et. al. | 6 | 2004 | 293 | 48.65 | 43.34 | CLBP | 4 | Valdecoxib | Placebo | NA |
| Skljarevski V et. al. | 7 | 2010 | 236 | 51.49 | 38.98 | CLBP | 13 | Duloxetine | Placebo | NA |
| Dakin P et. al. | 8 | 2020 | 282 | 56.75 | 41.84 | CLBP | 16 | Placebo | Fasinumab | NA |
| Katz N et. al. | 9 | 2011 | 217 | 51.06 | 45.62 | CLBP | 12 | Tanezumab | Naproxen | Placebo |
| Kivitz AJ et. al. | 10 | 2013 | 820 | 51.88 | 46.10 | CLBP | 16 | Placebo | Tanezumab | Naproxe |
| Sanga P et. al. | 11 | 2016 | 153 | 53.39 | 44.44 | CLBP | 12 | Placebo | Fulranumab | NA |
| Pallay RM et. al. | 12 | 2004 | 219 | 51.33 | 35.62 | CLBP | / | Placebo | Etoricoxib | NA |
| Tiseo PJ et. al. | 13 | 2014 | 104 | 44.90 | 47.12 | ALBP | 4 | Placebo | Fasinumab | NA |
| Friedman BW et. al. | 14 | 2015 | 323 | 38.67 | 51.08 | ALBP | 1 | Naproxen | Naproxen combined with cyclobenzaprine | Naproxen combined with Acetaminophen |
| Bedaiwi MK et. al. | 15 | 2016 | 50 | 40.3 | 50.00 | CLBP | 4 | Acetaminophen | Celecoxib | NA |
| Buynak R et. al. | 16 | 2010 | 965 | 49.93 | 42.07 | CLBP | 12 | Placebo | Tapentadol | Oxycodone |
| Kanayama M et. al. | 17 | 2005 | 40 | 32.75 | 50.00 | CLBP | 2 | Sarpogrelate | Diclofenac | NA |
| Markman JD et. al. | 18 | 2020 | 1418 | 48.77 | 44.08 | CLBP | 16 | Placebo | Tanezumab | Tramadol |
| Hale M et. al. | 19 | 2010 | 266 | 48.6 | 49.62 | CLBP | 12 | Hydromorphone | Placebo | NA |
| Miki K et. al. | 20 | 2018 | 70 | 68.1 | 18.57 | ALBP | 4 | Acetaminophen | Loxoprofen | NA |
| Konno S et. al. | 21 | 2016 | 456 | 58.91 | 48.03 | CLBP | 14 | Placebo | Duloxetine 60mg | NA |
| Peloso PM et. al. | 22 | 2004 | 336 | 57.5 | 37.50 | CLBP | 14 | Tramadol combined with Acetaminophen | Placebo | NA |
| Perrot S et. al. | 23 | 2006 | 119 | 55.29 | 42.02 | SALBP | 2 | Tramadol combined with Acetaminophen | Tramadol | NA |
| Rauck RL et. al. | 24 | 2014 | 302 | 50.6 | 44.70 | CLBP | 12 | Hydrocodone | Placebo | NA |
| Biondi D et. al. | 25 | 2013 | 585 | 45 | 49.91 | ALBP | 2 | Tapentadol | oxycodone | NA |
| Stein D et. al. | 26 | 1996 | 39 | 36.48 | 64.10 | ALBP | 6 | Amitriptyline | Acetaminophen | NA |
| Berry H et. al. | 27 | 1988 | 105 | 42.49 | 55.24 | ALBP | 1 | Tizanidine combined with Ibuprofen | ibuprofen | NA |
| Baron R et. al. | 28 | 2016 | 258 | 58.2 | 37.60 | CLBP | 12 | oxycodone | Tapentadol | NA |
| Ruoff GE et. al. | 29 | 2003 | 318 | 54.85 | 36.79 | CLBP | 13 | Tramadol combined with Acetaminophen | Placebo | NA |
| Birbara CA et. al. | 30 | 2003 | 208 | 51.63 | 40.87 | CLBP | 12 | Placebo | Etoricoxib | NA |

NR: Not reported; NA: Not applicable; ALBP: Acute low back pain; SALBP: Sub-acute low back pain; CLBP: Chronic low back pain.

**Supplemental table 2.** Methodological quality and risk of bias evaluation of included study.

| Author | No. | Sequence generation | Allocation concealment | Blinding | Incomplete outcome data | Selective outcome reporting | Other source of bias |
| --- | --- | --- | --- | --- | --- | --- | --- |
| Skljarevski V et. al. | 1 | L | L | L | L | L | U |
| Rauck RL et. al. | 2 | L | L | L | L | L | U |
| Pareek A et. al. | 3 | U | L | L | L | L | U |
| Gottlieb M et. al. | 4 | H | L | L | L | L | U |
| Skljarevski V et. al. | 5 | U | U | L | L | L | U |
| Coats TL et. al. | 6 | L | U | L | L | L | U |
| Skljarevski V et. al. | 7 | U | U | L | L | L | U |
| Dakin P et. al. | 8 | L | L | L | L | L | U |
| Katz N et. al. | 9 | U | U | L | L | L | U |
| Kivitz AJ et. al. | 10 | U | U | L | L | L | U |
| Sanga P et. al. | 11 | L | U | L | L | L | U |
| Pallay RM et. al. | 12 | L | L | L | L | L | U |
| Tiseo PJ et. al. | 13 | U | U | L | L | L | U |
| Friedman BW et. al. | 14 | L | L | L | L | L | U |
| Bedaiwi MK et. al. | 15 | L | L | L | L | L | U |
| Buynak R et. al. | 16 | L | U | L | L | L | U |
| Kanayama M et. al. | 17 | L | U | U | L | L | U |
| Markman JD et. al. | 18 | L | L | L | L | L | U |
| Hale M et. al. | 19 | L | U | L | L | L | U |
| Miki K et. al. | 20 | L | U | L | L | L | U |
| Konno S et. al. | 21 | L | L | L | L | L | U |
| Peloso PM et. al. | 22 | L | L | L | L | L | U |
| Perrot S et. al. | 23 | L | L | L | L | L | U |
| Rauck RL et. al. | 24 | U | L | L | L | L | U |
| Biondi D et. al. | 25 | L | L | L | L | L | U |
| Stein D et. al. | 26 | U | L | L | L | L | U |
| Berry H et. al. | 27 | U | U | L | L | L | U |
| Baron R et. al. | 28 | U | H | L | L | L | U |
| Ruoff GE et. al. | 29 | L | U | L | L | L | U |
| Birbara CA et. al. | 30 | L | L | L | L | L | U |

L: low risk of bias. U: unclear risk of bias. H: high risk of bias.

**Supplemental table 3**. Evidence contribution diagram for the pain relief network. The numbers are to the weight attached to each direct summary effect (horizontal axis) for the estimation of each network summary effects (vertical axis) as percentages. (A: Pla; B: ADP; C: ANGF; D: AP; E: cAPWO; F: NSAID; G: cNSMRS; H: SOP; I: WOP.)

|  |  |  | **Direct comparisions in the network** | | | | | | | | | | | | | |
| --- | --- | --- | --- | --- | --- | --- | --- | --- | --- | --- | --- | --- | --- | --- | --- | --- |
|  |  |  | A vs B | A vs C | A vs E | A vs F | A vs H | A vs I | B vs D | B vs F | C vs F | C vs I | D vs F | E vs I | F vs G | H vs I |
| **Network meta-analysis estimates** | Mixed estimates | |  |  |  |  |  |  |  |  |  |  |  |  |  |  |
|  |  | A vs B | 96.6 |  |  | 1.3 |  | 0.1 | 0.4 | 1.0 | 0.1 | 0.4 |  |  |  |  |
|  |  | A vs C | 0.1 | 3.9 |  | 0.1 | 12.1 | 20.5 |  | 0.1 | 0.2 | 38.7 |  | 6.0 |  | 12.1 |
|  |  | A vs E |  | 0.8 | 71.7 |  | 4.0 | 6.8 |  |  |  | 0.8 |  | 11.7 |  | 4.0 |
|  |  | A vs F | 34.4 | 0.1 | 0.2 | 16.7 |  | 0.7 | 9.5 | 24.9 | 1.5 | 1.4 |  | 0.2 |  | 0.4 |
|  |  | A vs H |  | 1.3 | 3.4 |  | 62.6 | 11.5 |  |  | 0.1 | 1.3 |  | 3.4 |  | 16.3 |
|  |  | A vs I | 0.1 | 3.7 | 9.8 | 0.1 | 19.6 | 33.1 |  | 0.1 | 0.2 | 3.9 |  | 9.8 |  | 19.6 |
|  |  | B vs D | 8.7 | 0.1 | 0.1 | 8.0 | 0.2 | 0.3 | 46.7 | 12.7 | 0.7 | 0.7 |  | 0.1 |  | 0.2 |
|  |  | B vs F | 21.0 | 0.2 | 0.2 | 19.2 | 0.5 | 0.8 | 11.7 | 30.6 | 1.7 |  |  | 0.2 |  | 0.5 |
|  |  | C vs F | 15.9 | 2.2 | 3.4 | 7.7 | 6.7 | 11.4 | 4.4 | 11.5 | 0.8 |  |  | 3.4 |  | 6.7 |
|  |  | C vs I | 0.1 | 3.4 | 0.6 | 0.1 | 1.1 | 1.9 |  | 0.1 | 0.2 | 90.8 |  | 0.6 |  | 1.1 |
|  |  | D vs F | 11.7 | 0.1 | 0.1 | 10.7 | 0.3 | 0.5 | 28.7 | 17.1 | 1.0 | 0.9 |  | 0.7 |  | 0.3 |
|  |  | E vs I | 0.1 | 2.3 | 35.4 |  | 12.3 | 20.7 |  | 0.1 | 0.1 | 2.4 |  | 14.4 |  | 12.3 |
|  |  | F vs G |  |  |  |  |  |  |  |  |  |  |  |  | 100.0 |  |
|  |  | H vs I | 0.1 | 2.4 | 6.3 |  | 30.2 | 21.4 |  | 0.1 | 0.1 | 2.5 |  | 6.3 |  | 30.4 |
|  | Indirect estimates | |  |  |  |  |  |  |  |  |  |  |  |  |  |  |
|  |  | A vs D | 38.9 |  | 0.1 | 5.9 | 0.2 | 0.3 | 30.9 | 8.0 | 0.5 | 0.5 | 14.5 | 0.1 |  | 0.2 |
|  |  | A vs G | 22.6 | 0.1 | 0.1 | 10.9 | 0.3 | 0.5 | 6.2 | 16.3 | 1.0 | 0.9 | 6.2 | 0.1 | 34.5 | 0.3 |
|  |  | B vs C | 29.4 | 2.7 | 4.2 | 0.3 | 8.5 | 14.3 | 0.1 | 0.4 | 0.2 | 27.0 | 0.1 | 4.2 |  | 8.5 |
|  |  | B vs E | 44.5 | 0.4 | 38.8 | 0.6 | 2.2 | 3.7 | 0.2 | 0.5 | 0.1 | 0.5 | 0.2 | 6.3 |  | 2.2 |
|  |  | B vs G | 12.8 | 0.1 | 0.2 | 11.8 | 0.3 | 0.5 | 7.0 | 18.8 | 1.1 | 1.0 | 7.2 | 0.2 | 38.8 | 0.3 |
|  |  | B vs H | 43.1 | 0.7 | 1.9 | 0.6 | 34.7 | 6.4 | 0.2 | 0.5 | 0.1 | 0.8 | 0.2 | 1.9 |  | 9.0 |
|  |  | B vs I | 39.1 | 2.2 | 5.8 | 0.5 | 11.7 | 19.8 | 0.2 | 0.5 | 0.2 | 2.4 | 0.2 | 5.8 |  | 11.7 |
|  |  | C vs D | 19.1 | 2.0 | 3.1 | 2.9 | 6.3 | 10.6 | 15.2 | 3.9 | 0.4 | 20.2 | 7.2 | 3.1 |  | 6.3 |
|  |  | C vs E | 0.1 | 2.9 | 25.0 | 0.1 | 8.2 | 13.8 |  | 0.1 | 0.2 | 31.8 |  | 9.8 |  | 8.2 |
|  |  | C vs G | 12.8 | 1.7 | 2.7 | 6.2 | 5.4 | 9.1 | 3.5 | 9.3 | 0.7 | 17.3 | 3.5 | 2.7 | 19.7 | 5.4 |
|  |  | C vs H | 0.1 | 3.0 | 3.9 | 0.1 | 20.1 | 13.1 |  | 0.1 | 0.2 | 36.3 |  | 3.9 |  | 19.3 |
|  |  | D vs E | 25.4 | 0.2 | 25.5 | 3.9 | 1.3 | 2.3 | 20.2 | 5.2 | 0.4 | 0.6 | 9.5 | 4.2 |  | 1.3 |
|  |  | D vs G | 7.4 | 0.1 | 0.1 | 6.8 | 0.2 | 0.3 | 18.2 | 10.8 | 0.6 | 0.6 | 18.2 | 0.1 | 36.5 | 0.2 |
|  |  | D vs H | 24.9 | 0.4 | 1.2 | 3.8 | 23.0 | 4.1 | 19.8 | 5.1 | 0.4 | 0.8 | 9.3 | 1.2 |  | 6.1 |
|  |  | D vs I | 23.3 | 1.5 | 4.0 | 3.5 | 8.0 | 13.5 | 18.6 | 4.8 | 0.4 | 1.9 | 8.7 | 4.0 |  | 8.0 |
|  |  | E vs F | 21.6 | 0.2 | 28.3 | 10.5 | 1.3 | 2.3 | 6.0 | 15.7 | 1.0 | 1.2 | 6.0 | 4.8 |  | 1.3 |
|  |  | E vs G | 16.3 | 0.2 | 21.3 | 7.9 | 1.0 | 1.7 | 4.5 | 11.8 | 0.7 | 0.9 | 4.5 | 3.6 | 24.8 | 1.0 |
|  |  | E vs H |  | 0.3 | 39.3 |  | 353.0 | 3.1 |  |  |  | 0.4 |  | 8.8 |  | 12.2 |
|  |  | F vs H | 21.1 | 0.4 | 1.3 | 10.2 | 25.3 | 4.3 | 5.8 | 15.3 | 0.9 | 1.4 | 5.8 | 1.3 |  | 6.9 |
|  |  | F vs I | 19.7 | 1.6 | 4.3 | 9.5 | 8.7 | 14.6 | 5.4 | 14.2 | 1.0 | 2.5 | 5.4 | 4.3 |  | 8.7 |
|  |  | G vs H | 15.9 | 0.3 | 1.0 | 7.7 | 19.2 | 3.2 | 4.4 | 11.5 | 0.7 | 1.0 | 4.4 | 1.0 |  | 5.2 |
|  |  | G vs I | 15.1 | 1.2 | 3.3 | 7.3 | 6.7 | 11.2 | 4.2 | 10.9 | 0.7 | 2.0 | 4.2 | 3.3 | 23.2 | 6.7 |
| Entire network | | | 18.0 | 1.2 | 8.9 | 5.3 | 9.9 | 8.0 | 7.8 | 7.9 | 0.5 | 8.6 | 5.4 | 3.5 | 8.5 | 6.3 |
| Included studies | | | 4 | 6 | 2 | 5 | 4 | 2 | 1 | 1 | 2 | 1 | 2 | 1 | 2 | 3 |

**Supplemental table 4**. Evidence contribution diagram for the function improvement network. The numbers are to the weight attached to each direct summary effect (horizontal axis) for the estimation of each network summary effects (vertical axis) as percentages. (A: Pla; B: ADP; C: ANGF; D: AP; E: cAPWO; F: NSAID; G: cNSMRS; H: SOP; I: WOP.)

|  |  |  | **Direct comparisions in the network** | | | | | | | | | | | |
| --- | --- | --- | --- | --- | --- | --- | --- | --- | --- | --- | --- | --- | --- | --- |
|  |  |  | A vs B | A vs C | A vs E | A vs F | A vs H | A vs I | B vs F | C vs F | C vs I | D vs F | F vs G | H vs I |
| **Network meta-analysis estimates** | Mixed estimates | |  |  |  |  |  |  |  |  |  |  |  |  |
|  |  | A vs B | 94.8 |  |  | 2.6 |  |  | 2.6 |  |  |  |  |  |
|  |  | A vs C |  | 3.8 |  | 0.2 | 0.1 | 47.7 |  | 0.3 | 47.8 |  |  | 0.1 |
|  |  | A vs E |  |  | 100.0 |  |  |  |  |  |  |  |  |  |
|  |  | A vs F | 10.0 |  |  | 78.0 |  | 0.6 | 10.0 | 0.7 | 0.6 |  |  |  |
|  |  | A vs H |  | 1.8 |  | 0.1 | 0.4 | 46.9 |  | 0.1 | 1.9 |  |  | 48.8 |
|  |  | A vs I |  | 3.5 |  | 0.2 | 0.2 | 92.0 |  | 0.2 | 3.7 |  |  | 0.2 |
|  |  | B vs F | 45.9 |  |  | 45.5 |  | 0.4 | 7.4 | 0.4 | 0.4 |  |  |  |
|  |  | C vs F | 3.7 |  |  | 29.0 | 0.1 | 30.3 | 3.7 | 0.4 | 30.3 |  |  | 0.1 |
|  |  | C vs I |  | 2.4 |  | 0.2 |  | 3.7 |  | 0.2 | 92.3 |  |  |  |
|  |  | D vs F |  | 3.5 |  |  |  |  |  |  |  | 100.0 |  |  |
|  |  | F vs G |  |  |  |  |  |  |  |  |  |  | 100.0 |  |
|  |  | H vs I |  |  |  |  | 0.7 | 0.7 |  |  |  |  |  | 98.6 |
|  | Indirect estimates | |  |  |  |  |  |  |  |  |  |  |  |  |
|  |  | A vs D | 5.3 |  |  | 44.0 |  | 0.3 | 5.3 | 0.4 | 0.3 | 47.0 |  |  |
|  |  | A vs G | 5.3 |  |  | 41.3 |  | 0.3 | 5.3 | 0.4 | 0.3 |  | 47.0 |  |
|  |  | B vs C | 33.0 | 2.5 |  | 0.7 | 0.1 | 31.2 | 0.9 | 0.2 | 31.3 |  |  | 0.1 |
|  |  | B vs D | 29.9 |  |  | 29.7 |  | 0.2 | 4.9 | 0.3 | 0.2 | 34.8 |  |  |
|  |  | B vs E | 48 |  | 49.3 | 1.3 |  |  | 1.3 | . |  |  |  |  |
|  |  | B vs G | 28.9 |  |  | 29.7 |  | 0.2 | 4.9 | 0.3 | 0.2 |  | 34.8 |  |
|  |  | B vs H | 31.9 | 1.2 |  | 0.8 | 0.3 | 31.2 | 0.9 | 0.1 | 1.3 |  |  | 32.5 |
|  |  | B vs I | 47.1 | 1.8 |  | 1.2 | 0.1 | 46.5 | 1.3 | 0.1 | 1.9 |  |  | 0.1 |
|  |  | C vs D | 2.8 | 1.8 |  | 21.8 |  | 22.7 | 2.8 | 0.3 | 22.8 | 24.9 |  |  |
|  |  | C vs E |  | 2.5 | 34.1 | 0.2 | 0.1 | 31.4 |  | 0.2 | 31.5 |  |  | 0.1 |
|  |  | C vs G | 2.8 | 1.8 |  | 21.8 |  | 22.7 | 2.8 | 0.3 | 22.8 |  | 24.9 |  |
|  |  | C vs H |  | 1.8 |  | 0.1 | 0.3 | 1.6 |  | 0.1 | 47.2 |  |  | 44.8 |
|  |  | D vs E | 3.6 |  | 32.0 | 28.1 |  | 0.2 | 3.6 | 0.2 | 0.2 | 32.0 |  |  |
|  |  | D vs G |  |  |  |  |  |  |  |  |  | 50.0 | 50.0 |  |
|  |  | D vs H | 2.7 | 0.9 |  | 21.2 | 0.2 | 22.8 | 2.7 | 0.2 | 1.1 | 24.2 |  | 24.0 |
|  |  | D vs I | 3.6 | 1.1 |  | 27.0 | 0.1 | 30.2 | 3.6 | 0.3 | 1.5 | 31.7 |  | 0.1 |
|  |  | E vs F | 5.3 |  | 47.0 | 41.3 |  | 0.3 | 5.3 | 0.4 | 0.3 |  |  |  |
|  |  | E vs G | 3.6 |  | 32.0 | 28.1 |  | 0.2 | 3.6 | 0.2 | 0.2 |  | 32.0 |  |
|  |  | E vs H |  | 1.2 | 33.0 | 0.1 | 0.3 | 31.4 |  | 1.3 | 1.3 |  |  | 32.7 |
|  |  | E vs I |  | 1.8 | 48.9 | 0.1 | 0.1 | 47.0 |  | 1.9 | 1.9 |  |  | 0.1 |
|  |  | F vs H | 3.6 | 1.1 |  | 27.8 | 0.3 | 30.1 | 3.6 | 1.5 | 1.5 |  |  | 31.6 |
|  |  | F vs I | 5.2 | 1.7 |  | 40.8 | 0.1 | 44.3 | 5.2 | 2.1 | 2.1 |  |  | 0.1 |
|  |  | G vs H | 2.7 | 0.9 |  | 21.2 | 0.2 | 22.8 | 2.7 | 1.1 | 1.1 |  | 24.4 | 24.0 |
|  |  | G vs I | 3.6 | 1.1 |  | 27.8 | 0.1 | 30.2 | 3.6 | 1.5 | 1.5 |  | 31.7 | 0.1 |
| Entire network | | | 10.7 | 1.1 | 9.4 | 18.6 | 0.1 | 20.0 | 2.6 | 0.2 | 9.4 | 9.4 | 9.4 | 9.3 |
| Included studies | | | 4 | 6 | 2 | 5 | 2 | 1 | 1 | 2 | 1 | 2 | 4 | 1 |

**Supplemental table 5**. Evidence contribution diagram for the TEAEs network. The numbers are to the weight attached to each direct summary effect (horizontal axis) for the estimation of each network summary effects (vertical axis) as percentages. (A: Pla; B: ADP; C: ANGF; D: AP; E: cAPWO; F: NSAID; G: cNSMRS; H: SOP; I: WOP.)

|  |  |  | **Direct comparisions in the network** | | | | | | | | | |
| --- | --- | --- | --- | --- | --- | --- | --- | --- | --- | --- | --- | --- |
|  |  |  | A vs B | A vs C | A vs F | A vs H | A vs I | C vs I | D vs F | E vs I | F vs G | H vs I |
| **Network meta-analysis estimates** | Mixed estimates | |  |  |  |  |  |  |  |  |  |  |
|  |  | A vs B | 100.0 |  |  |  |  |  |  |  |  |  |
|  |  | A vs C |  | 50.5 |  | 1.7 | 22.2 | 23.9 |  |  |  | 1.7 |
|  |  | A vs F |  |  | 100.0 |  |  |  |  |  |  |  |
|  |  | A vs H |  | 7.2 |  | 29.4 | 24.4 | 7.2 |  |  |  | 31.7 |
|  |  | A vs I |  | 16.9 |  | 4.4 | 57.3 | 16.9 |  |  |  | 4.4 |
|  |  | C vs I |  | 31.0 |  | 2.2 | 28.7 | 35.9 |  |  |  | 2.2 |
|  |  | D vs F |  |  | 0.1 |  |  |  | 99.8 |  |  |  |
|  |  | E vs I |  |  |  |  |  |  |  | 100.0 |  |  |
|  |  | F vs G |  |  |  |  |  |  |  |  | 100.0 |  |
|  |  | H vs I |  | 6.4 |  | 27.9 | 21.6 | 6.4 |  |  |  | 37.8 |
|  | Indirect estimates | |  |  |  |  |  |  |  |  |  |  |
|  |  | A vs D |  |  | 50.0 |  |  |  | 50.0 |  |  |  |
|  |  | A vs G |  | 9.5 |  | 2.5 | 32.1 | 9.5 |  | 44.0 |  | 2.5 |
|  |  | A vs E |  |  | 50.0 |  |  |  |  |  | 50.0 |  |
|  |  | B vs C | 42.7 | 28.9 |  | 1.0 | 12.7 | 13.7 |  |  |  | 1.0 |
|  |  | B vs D | 33.3 |  | 33.3 |  |  |  | 33.3 |  |  |  |
|  |  | B vs E | 30.6 | 6.6 |  | 1.7 | 22.3 | 6.6 |  | 30.6 |  | 1.7 |
|  |  | B vs F | 50 |  | 50.0 |  |  |  |  |  |  |  |
|  |  | B vs G | 33.3 |  | 33.3 |  |  |  |  |  | 33.3 |  |
|  |  | B vs H | 37.9 | 4.5 |  | 18.3 | 15.2 | 4.5 |  |  |  | 19.7 |
|  |  | B vs I | 44 | 9.5 |  | 2.5 | 32.1 | 9.6 |  |  |  | 2.5 |
|  |  | C vs D |  | 20.3 | 29.9 | 0.7 | 8.9 | 9.6 | 29.9 |  |  | 0.7 |
|  |  | C vs E |  | 18.6 |  | 1.3 | 17.2 | 21.5 |  | 40.1 |  | 1.3 |
|  |  | C vs F |  | 28.9 | 42.7 | 1.0 | 12.7 | 13.7 |  |  |  | 1.0 |
|  |  | C vs G |  | 20.3 | 29.9 | 0.7 | 8.9 | 9.6 |  |  | 29.9 | 0.7 |
|  |  | C vs H |  | 26.7 |  | 21.8 | 4.8 | 20.9 |  |  |  | 25.8 |
|  |  | D vs E |  | 5.0 | 23.4 | 1.3 | 17.0 | 5.0 | 23.4 | 23.4 |  | 1.3 |
|  |  | D vs G |  |  |  |  |  |  | 49.9 |  | 50.0 |  |
|  |  | D vs H |  | 3.3 | 27.5 | 13.2 | 11.0 | 3.3 | 27.5 |  |  | 14.3 |
|  |  | D vs I |  | 6.6 | 30.6 | 1.7 | 22.3 | 6.6 | 30.6 |  |  | 1.7 |
|  |  | E vs F |  | 6.6 | 30.6 | 1.7 | 22.3 | 6.6 |  | 30.6 |  | 1.7 |
|  |  | E vs G |  | 5.0 | 23.4 | 1.3 | 17.0 | 5.0 |  | 39.4 | 23.4 | 1.3 |
|  |  | E vs H |  | 3.8 |  | 16.9 | 13.0 | 3.8 |  | 39.6 |  | 22.8 |
|  |  | F vs H |  | 4.5 | 37.9 | 18.3 | 15.2 | 4.5 |  |  |  | 19.7 |
|  |  | F vs I |  | 9.5 | 44.0 | 2.5 | 32.1 | 9.5 |  |  |  | 2.5 |
|  |  | G vs H |  | 3.3 | 27.5 | 13.2 | 11.0 | 3.3 |  |  | 27.5 | 14.3 |
|  |  | G vs I |  | 6.6 | 30.6 | 1.7 | 22.3 | 6.6 |  |  | 30.6 | 1.7 |
| Entire network | | | 9.3 | 9.1 | 21.0 | 5.2 | 14.5 | 7.1 | 9.3 | 9.3 | 9.3 | 5.9 |
| Included studies | | | 3 | 4 | 3 | 4 | 2 | 1 | 1 | 1 | 4 | 3 |

**Supplemental table 6**. Evidence contribution diagram for the SAEs network. The numbers are to the weight attached to each direct summary effect (horizontal axis) for the estimation of each network summary effects (vertical axis) as percentages. (A: Pla; B: ADP; C: ANGF; D: cAPWO; E: NSAID; F: cNSMRS; G: SOP; H: WOP.)

|  |  |  | **Direct comparisions in the network** | | | | | | | | | |  |
| --- | --- | --- | --- | --- | --- | --- | --- | --- | --- | --- | --- | --- | --- |
|  |  |  | A vs B | A vs C | A vs D | A vs E | A vs G | A vs H | C vs E | C vs H | D vs H | E vs F | G vs H |
| **Network meta-analysis estimates** | Mixed estimates | |  |  |  |  |  |  |  |  |  |  |  |
|  |  | A vs B | 99.9 |  |  |  |  |  |  |  |  |  |  |
|  |  | A vs C |  | 53.9 | 0.3 | 6.5 | 5.4 | 8.1 | 6.5 | 13.7 | 0.3 |  | 5.4 |
|  |  | A vs D |  | 5.2 | 43.9 | 0.6 | 6.3 | 9.4 | 0.6 | 5.9 | 21.6 |  | 6.3 |
|  |  | A vs E |  | 19.3 | 0.1 | 44.6 | 1.8 | 2.9 | 24.2 | 4.9 | 0.1 |  | 1.9 |
|  |  | A vs G |  | 7.9 | 0.5 | 1.0 | 42.4 | 14.3 | 1.0 | 8.9 | 0.5 |  | 23.6 |
|  |  | A vs H |  | 14.9 | 0.9 | 1.8 | 18.1 | 26.8 | 1.8 | 16.7 | 0.9 |  | 18.1 |
|  |  | C vs E |  | 26.0 | 0.1 | 32.6 | 2.6 | 3.9 | 25.5 | 6.6 | 0.1 |  | 2.6 |
|  |  | C vs H |  | 26.7 | 0.6 | 3.2 | 11.8 | 17.5 | 3.2 | 24.7 | 9.0 |  | 11.8 |
|  |  | D vs H |  | 7.8 | 32.1 | 0.9 | 9.4 | 14.0 | 0.9 | 8.7 | 16.8 |  | 2.4 |
|  |  | E vs F |  |  |  | 0.1 |  |  | 0.1 |  |  | 99.6 |  |
|  |  | G vs H |  | 7.9 | 0.5 | 0.9 | 23.4 | 14.1 | 0.9 | 8.8 | 0.5 |  | 1.0 |
|  | Indirect estimates | |  |  |  |  |  |  |  |  |  |  |  |
|  |  | A vs F |  | 11.4 | 0.1 | 26.4 | 1.2 | 1.7 | 14.3 | 2.9 | 0.1 | 40.7 | 1.2 |
|  |  | B vs C | 42.5 | 31.0 | 0.2 | 3.7 | 3.1 | 4.6 | 3.7 | 7.9 | 0.2 |  | 3.1 |
|  |  | B vs D | 28.6 | 3.2 | 26.5 | 0.4 | 3.8 | 5.7 | 0.4 | 3.5 | 13.1 |  | 3.8 |
|  |  | B vs E | 40.8 | 11.4 | 0.1 | 23.4 | 1.1 | 1.7 | 14.3 | 2.9 | 0.1 |  | 1.2 |
|  |  | B vs G | 39.8 | 4.8 | 0.3 | 0.6 | 25.5 | 8.6 | 0.6 | 5.4 | 0.3 |  | 14..3 |
|  |  | B vs H | 38.5 | 9.2 | 0.5 | 1.1 | 11.1 | 16.5 | 1.1 | 10.3 | 0.6 |  | 11.1 |
|  |  | B vs F | 29.0 | 8.1 |  | 18.8 | 0.8 | 1.2 | 10.2 | 2.1 |  | 28.9 | 0.8 |
|  |  | C vs D |  | 30.0 | 30.9 | 3.6 | 1.1 | 1.6 | 3.6 | 12.7 | 15.4 |  | 1.1 |
|  |  | C vs G |  | 27.7 | 0.2 | 3.3 | 26.0 | 4.9 | 3.3 | 14.6 | 0.2 |  | 19.7 |
|  |  | C vs F |  | 16.5 | 0.1 | 20.6 | 1.6 | 2.4 | 16.1 | 4.2 | 0.1 | 36.7 | 1.7 |
|  |  | D vs E |  | 7.7 | 25.7 | 24.6 | 2.6 | 3.9 | 13.9 | 6.2 | 12.7 |  | 2.6 |
|  |  | D vs G |  | 1.9 | 30.6 | 0.2 | 25.1 | 3.4 | 0.2 | 2.1 | 15.5 |  | 21.0 |
|  |  | D vs F |  | 5.6 | 18.6 | 17.7 | 1.9 | 2.8 | 10.0 | 4.5 | 9.2 | 27.8 | 1.9 |
|  |  | E vs G |  | 6.3 | . | 24.8 | 24.0 | 6.8 | 14.3 | 8.1 | 0.2 |  | 15.1 |
|  |  | E vs H |  | 1.8 | 0.6 | 26.8 | 11.3 | 16.7 | 16.4 | 14.6 | 0.6 |  | 11.3 |
|  |  | G vs F |  | 4.5 | 0.2 | 17.8 | 17.3 | 4.9 | 10.5 | 5.8 | 0.2 | 28.1 | 10.9 |
|  |  | H vs F |  | 1.2 | 0.4 | 18.7 | 7.9 | 11.7 | 11.5 | 10.2 | 0.4 | 30.1 | 7.9 |
| Entire network | | | 11.3 | 11.6 | 7.7 | 12.9 | 10.0 | 7.2 | 8.1 | 7.6 | 4.0 | 11.3 | 8.4 |
| Included studies | | | 4 | 6 | 2 | 4 | 4 | 2 | 2 | 1 | 1 | 1 | 3 |

**Supplemental table 7**. The league plots of subgroup efficacy analysis. Pain relief (Red) and function improvement (Blue). (From the top left to the bottom right, higher comparator vs lower comparator, SMD with 95% CI.)

| **ANGF** | -0.80 (-1.79,0.19) | 0.07 (-1.37,1.51) | -0.73 (-2.30,0.83) | -0.82 (-1.98,0.35) | 0.19 (-1.20,1.58) | -1.00 (-3.20,1.20) | -0.99 (-1.80,-0.18) |
| --- | --- | --- | --- | --- | --- | --- | --- |
| 0.40 (-0.52,1.32) | **NSAID** | 0.87 (-0.66,2.41) | 0.07 (-1.48,1.61) | -0.02 (-1.07,1.04) | 0.99 (-0.41,2.39) | -0.20 (-2.17,1.76) | -0.19 (-0.96,0.58) |
| 0.52 (-0.63,1.68) | 0.12 (-1.09,1.33) | **WOP** | -0.81 (-2.72,1.10) | -0.89 (-2.50,0.72) | 0.12 (-1.31,1.54) | -1.08 (-3.57,1.42) | -1.06 (-2.43,0.30) |
| 0.61 (-0.85,2.06) | 0.21 (-1.23,1.64) | 0.09 (-1.51,1.69) | **cAPWO** | -0.08 (-1.68,1.51) | 0.92 (-0.87,2.71) | -0.27 (-2.76,2.23) | -0.25 (-1.59,1.08) |
| 0.77 (-0.32,1.85) | 0.36 (-0.62,1.35) | 0.24 (-1.04,1.53) | 0.16 (-1.33,1.64) | **ADP** | 1.01 (-0.46,2.47) | -0.18 (-2.42,2.05) | -0.17 (-1.04,0.69) |
| 0.69 (-0.40,1.77) | 0.28 (-0.80,1.37) | 0.16 (-0.86,1.19) | 0.08 (-1.42,1.57) | -0.08 (-1.23,1.07) | **SOP** | -1.19 (-3.61,1.22) | -1.18 (-2.37,0.01) |
| 1.13 (-0.93,3.19) | 0.73 (-1.12,2.57) | 0.61 (-1.60,2.81) | 0.52 (-1.82,2.86) | 0.36 (-1.73,2.46) | 0.44 (-1.70,2.58) | **AP** | 0.01 (-2.10,2.12) |
| 1.04 (0.29,1.79) | 0.64 (-0.08,1.35) | 0.52 (-0.49,1.52) | 0.43 (-0.82,1.68) | 0.27 (-0.53,1.08) | 0.35 (-0.47,1.18) | -0.09 (-2.07,1.89) | **Pla** |

**Supplemental table 8**. The league plots of subgroup safety analysis. TEAEs (Red) and SAEs (Blue). (From the top left to the bottom right, higher comparator vs lower comparator, RR with 95% CI.)

| **ANGF** | 1.59 (0.55,4.60) | 1.01 (0.05,19.56) | 3.22 (0.82,12.67) | 2.07 (0.70,6.15) | 1.16 (0.32,4.19) | 1.02 (0.47,2.19) |
| --- | --- | --- | --- | --- | --- | --- |
| 0.75 (0.58,0.97) | **cAPWO** | 0.64 (0.03,12.89) | 2.02 (0.48,8.53) | 1.30 (0.49,3.41) | 0.73 (0.17,3.19) | 0.64 (0.25,1.63) |
| 0.91 (0.74,1.12) | 1.22 (0.95,1.57) | **WOP** | 3.18 (0.15,68.63) | 2.04 (0.10,40.43) | 1.15 (0.05,25.48) | 1.01 (0.06,17.56) |
| 0.97 (0.81,1.18) | 1.30 (1.02,1.65) | 1.07 (0.89,1.29) | **ADP** | 0.64 (0.16,2.64) | 0.36 (0.07,1.85) | 0.32 (0.10,0.98) |
| 1.00 (0.81,1.23) | 1.33 (1.03,1.72) | 1.09 (0.89,1.34) | 1.02 (0.85,1.24) | **SOP** | 0.56 (0.13,2.42) | 0.49 (0.21,1.17) |
| 1.22 (0.97,1.53) | 1.62 (1.23,2.13) | 1.33 (1.06,1.68) | 1.25 (1.01,1.55) | 1.22 (0.97,1.53) | **NSAID** | 0.88 (0.26,2.92) |
| 1.12 (0.90,1.40) | 1.01 (0.81,1.25) | 1.52 (1.15,2.01) | 1.14 (1.01,1.29) | 1.48 (1.20,1.83) | 1.11 (0.96,1.29) | **Pla** |

**Supplemental table 9**. The league plots of the second subgroup efficacy analysis. Pain relief (Red) and function improvement (Blue). (From the top left to the bottom right, higher comparator vs lower comparator, SMD with 95% CI.)

| **Tanezumab** | -0.67 (-4.79,3.44) | -1.10 (-5.21,3.00) | -1.22 (-3.27,0.84) |
| --- | --- | --- | --- |
| 1.02 (-3.16,5.20) | **Fasinumab** | -0.43 (-5.46,4.60) | -0.54 (-4.10,3.02) |
| 1.25 (-2.92,5.43) | 0.23 (-4.88,5.34) | **Fulranumab** | -0.11 (-3.67,3.44) |
| 1.30 (-0.79,3.39) | 0.28 (-3.34,3.90) | 0.05 (-3.57,3.66) | **Placebo** |

**Supplemental table 10**. The league plots of the second subgroup safety analysis. TEAEs (Red) and SAEs (Blue). (From the top left to the bottom right, higher comparator vs lower comparator, RR with 95% CI.)

| **Tanezumab** | 0.82 (0.10,7.02) | 1.08 (0.17,6.86) | 1.10 (0.34,3.50) |
| --- | --- | --- | --- |
| 1.09 (0.80,1.50) | **Fasinumab** | 1.32 (0.13,13.20) | 1.33 (0.22,8.09) |
| 1.05 (0.87,1.26) | 0.96 (0.69,1.34) | **Fulranumab** | 1.01 (0.24,4.26) |
| 1.18 (1.06,1.30) | 1.08 (0.80,1.45) | 1.12 (0.96,1.31) | **Placebo** |
